# Optimization of water and fertilizer regime for greenhouse radish using BBD response surface method coupled with NSGA-II algorithm

**DOI:** 10.3389/fpls.2025.1651490

**Published:** 2026-08-31

**Authors:** Yurong Wei, Pengrui Ai, Yingjie Ma, Yun Jiu

**Affiliations:** 1 College of Water Resources and Civil Engineering, Xinjiang Agricultural University, Urumqi, China; 2 Xinjiang Key Laboratory of Water, Urumqi, China

**Keywords:** greenhouse white radish, water and fertilizer coupling, response surface method, NSGA-II algorithm, vitamin C, soluble sugar

## Abstract

**Objective:**

To address the issue of yield-quality imbalance caused by the low water-fertilizer synergy efficiency of white radish in greenhouses in the cold region of northern Xinjiang, this study aimed to reveal the seasonal specific interaction mechanisms of irrigation amount (W), nitrogen application rate (N), and potassium application rate (K), and to construct a multi-objective optimized water-fertilizer management model.

**Methods:**

Experiments were conducted in summer and winter based on the Box - Behnken design. The interaction effects of water and fertilizers were analyzed through the response surface model, and the multi - objective collaborative optimization of yield, soluble sugar, and vitamin C was achieved by combining with the NSGA - II algorithm.

**Results:**

There were significant seasonal differences. In summer, the available potassium concentration in the 10 - cm soil layer (206.98 mg/kg) under the W2K1N2 treatment was 83.4% higher than that in the deeper layer, and the surface accumulation rate of ammonium nitrogen reached 33.0% in winter. Under the coordinated regulation of water, potassium, and nitrogen, the above - ground dry matter mass under the W2K1N2 treatment in summer increased by 32.0% compared with the control, while the stem diameter in winter decreased by 8.6% due to low - temperature inhibition. The main effect of potassium fertilizer was significant in summer (p < 0.05), and nitrogen was the yield - limiting factor in winter, which confirmed the rule of “potassium promotes quality, nitrogen promotes yield”. Season - specific water and fertilizer schemes were optimized based on the BBD - NSGA - II algorithm. In summer (W: 19.96 mm, K: 123.17 kg/hm², N: 198 kg/hm²), the simulated yield reached 151.55 t/hm², an increase of 93.1% compared with the control, and the soluble sugar increased by 18.9%. In winter (W: 28.03 mm, K: 182.51 kg/hm², N: 296.91 kg/hm²), the yield was 153.21 t/hm², an increase of 65.5% compared with the control, and the soluble sugar and vitamin C increased by 69.4% and 314.4% respectively.

**Conclusions:**

This optimization scheme provides a scientific basis for the efficient production of greenhouse white radishes, which can significantly improve the physiological indicators, yield, and quality of crops, and optimize the resource utilization efficiency. The cross - season water and fertilizer optimization scheme based on BBD - NSGA - II can achieve the coordinated improvement of yield and quality of greenhouse white radishes and the efficient use of resources, providing a theoretical basis and an intelligent decision - making paradigm for the precise regulation of greenhouse vegetables in cold regions.

## 1 Introduction

White radish belongs to root-tuber crops and is a plant of the genus Raphanus in the Brassicaceae family (Sun et al., 2024). It is widely used in both diet and traditional Chinese medicine and has a cultivation history of over a thousand years in China. As of 2021, the harvested area of radishes in China was 395,500 ha accounting for 36.09% of the global radish harvested area. Greenhouses belong to a high-input and high-income agricultural cultivation system (Shi et al., 2019). To ensure high yields farmers in greenhouses apply far more water and fertilizers than for field crops, Youdaoplaceholder0 in extremely low water and fertilizer use efficiency for greenhouse crops (Xing et al., 2015).

Water and fertilizers are key factors affecting crop growth and yield (Ma et al., 2023; Chen et al., 2024). Appropriate water and fertilizer regulation is an important guarantee for improving crop yield and quality (Fan et al., 2024; Liu et al., 2024). Youdaoplaceholder0 for the greenhouse radish cultivation industry in Xinjiang exploring the effects of different water and fertilizer management strategies on greenhouse radishes is an urgent need for agricultural production in Xinjiang (Zhou et al., 2024).

### 1.1 Previous research progress

Water–fertilizer coupling modulates greenhouse crop physiology and has become a cornerstone of precision protected agriculture (Gonzalez-Dugo et al., 2010). Meta-analyses and multi-site experiments show that targeted combinations of irrigation and a single nutrient can shift quality trajectories: tomato soluble sugars rise 12.3–15.6% under moderate N regimes, whereas K supply governs reducing- and total-sugar accumulation in flue-cured tobacco (Yin et al., 2012; Prajapati et al., 2012). In cucumber, 360 kg N ha^−1^ synchronized with 80% ETc irrigation elevates marketable yield (Wang et al., 2019), water-use efficiency and fruit sensory attributes without extra water input. Synergistic K-water management delivers simultaneous gains in productivity and resource efficiency. Tuber crops respond to optimized irrigation–potassium schedules with higher fresh mass, water productivity and whole-plant K accumulation (Zhang et al., 2022; Šustr et al., 2019). In humid subtropical orchards, slight K deficit under regulated deficit irrigation enhances kiwifruit firmness and nutritional density, offering a strategic lever for quality-oriented fertilization (Cui et al., 2023).

Methodological diversity characterizes current coupling research. Factorial quadratic-orthogonal rotation designs have been deployed to identify K-water-N formulations that maximize strawberry consumer acceptance (Yang et al., 2023). Multi-criteria decision frameworks combining entropy-weighted TOPSIS integrate water conservation, yield, profit and environmental targets to select optimum regimes for winter wheat (He et al., 2024). Four-dimensional quadratic-orthogonal models coupled with fuzzy-certainty evaluation have revealed seasonal optima (1978 m³ H_2_O, 482 kg N, 104 kg P_2_O_5_, 181 kg K_2_O ha^−1^) that reconcile yield with nutrient-use efficiency. Adaptive neuro-fuzzy inference systems (ANFIS) trained on N-P-K-water datasets accurately predict crop response surfaces and minimize input cost (Kuzman et al., 2021; Rudnick et al., 2013). Despite these advances, most investigations address binary nutrient–water interactions; quantitative insight into N-K-water ternary synergy remains fragmentary. Two-dimensional response surfaces introduce substantial uncertainty when extrapolated to three-factor space. Although quadratic-orthogonal designs can, in principle, handle multiple factors, treatment numbers increase exponentially, inflating cost and complicating non-linear interpretation. Entropy-weighted TOPSIS depends on subjective index weights, obscuring dynamic thresholds, whereas machine-learning approaches demand large training populations and exhibit limited transferability across sites and seasons.

### 1.2 Research entry point

To address the above issues, this study uses greenhouse white radishes as the test material. The Box - Behnken Design (BBD) response surface model combined with the Non - dominated Sorting Genetic Algorithm II (NSGA - II) is employed to optimize the experimental design. The irrigation and fertilization systems suitable for greenhouse white radishes in summer and winter are simulated to achieve the optimal irrigation and fertilization system for greenhouse radishes in terms of yield and quality.

### 1.3 Key scientific problems to be solved

This study innovatively integrates the Box - Behnken Design (BBD) and the Non - dominated Sorting Genetic Algorithm II (NSGA - II) to construct a “mechanism - data” dual - driven optimization model. By repeating the central points, the robustness of the model is enhanced, enabling multi - objective global optimization of yield, quality, and resource efficiency on the Pareto front. Through the synergy of explicit response surface modeling and implicit genetic algorithm search, the model combines interpretability and global optimization ability, providing a more general analytical framework for the precise regulation of complex water - fertilizer systems. It also provides a reference for the planting and management of radishes in greenhouses in summer and winter in the cold regions of northern Xinjiang (Zhou et al., 2024).

### 1.4 Point of innovation

This study assumes that the effect of water fertilizer coupling regulation on the yield and quality of greenhouse white radish is equivalent to the synergistic effect of nitrogen and potassium. In this study, we assume that the nitrogen potassium water synergistic optimization model constructed can ensure the optimal nutritional state of greenhouse white radish during the critical stage of yield formation. Based on this assumption, the following research objectives were formulated: to determine the impact of water fertilizer coupling on physiological indicators and yield quality of greenhouse radish, providing reference for cross seasonal water fertilizer regulation; 2. to simulate the optimal irrigation and fertilization scheme through the BBD-NSGA II model system, and achieve precise synergy of yield quality resource efficiency of greenhouse white radish under greenhouse conditions.

## 2 Materials and methods

### 2.1 Overview of the study area

**Figure 1:**
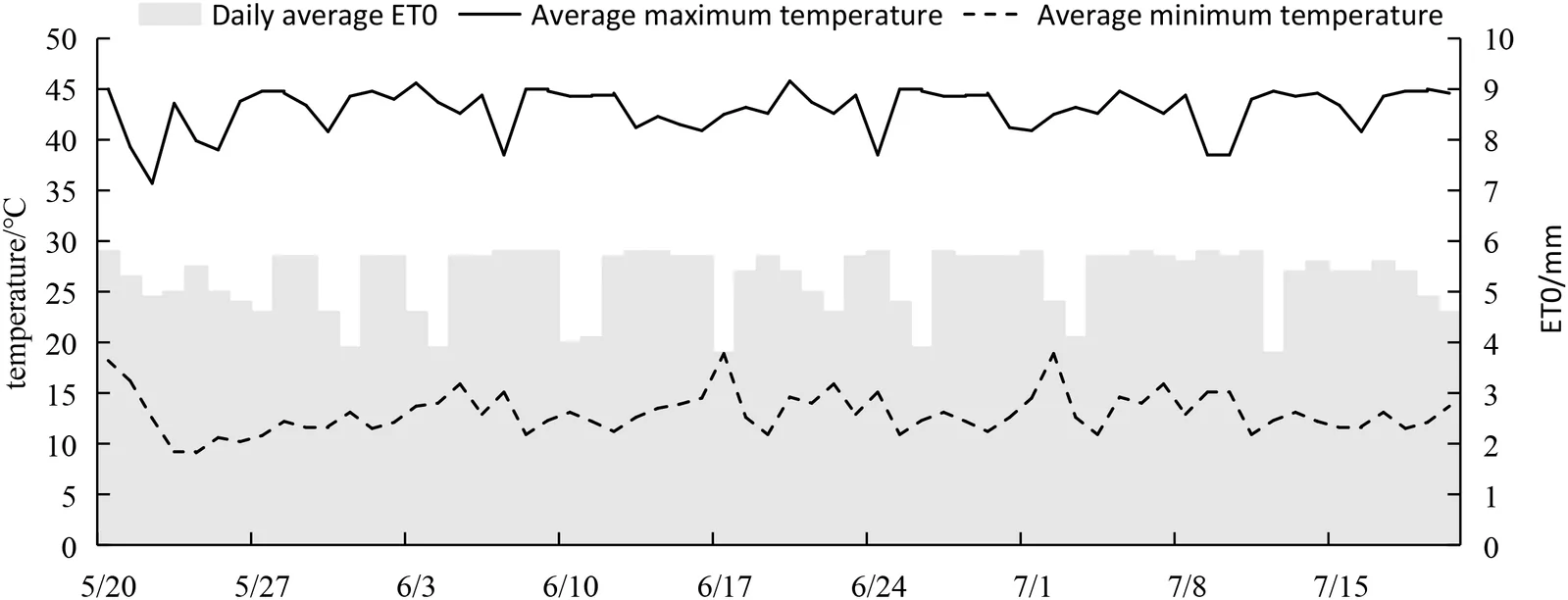
Daily average summer temperature and reference evapotranspiration data of the experimental site.

**Figure 2:**
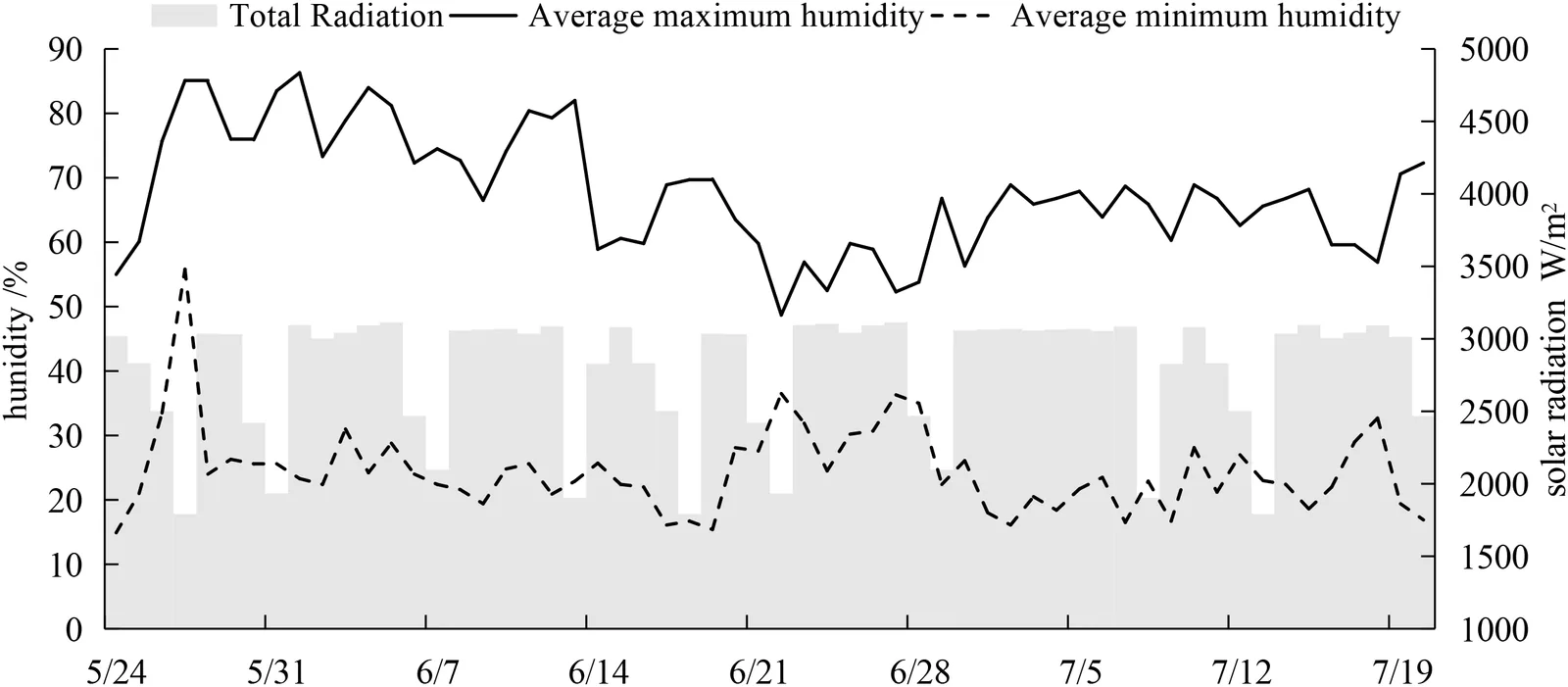
Daily average humidity and solar radiation data of the experimental site in summer.

**Figure 3:**
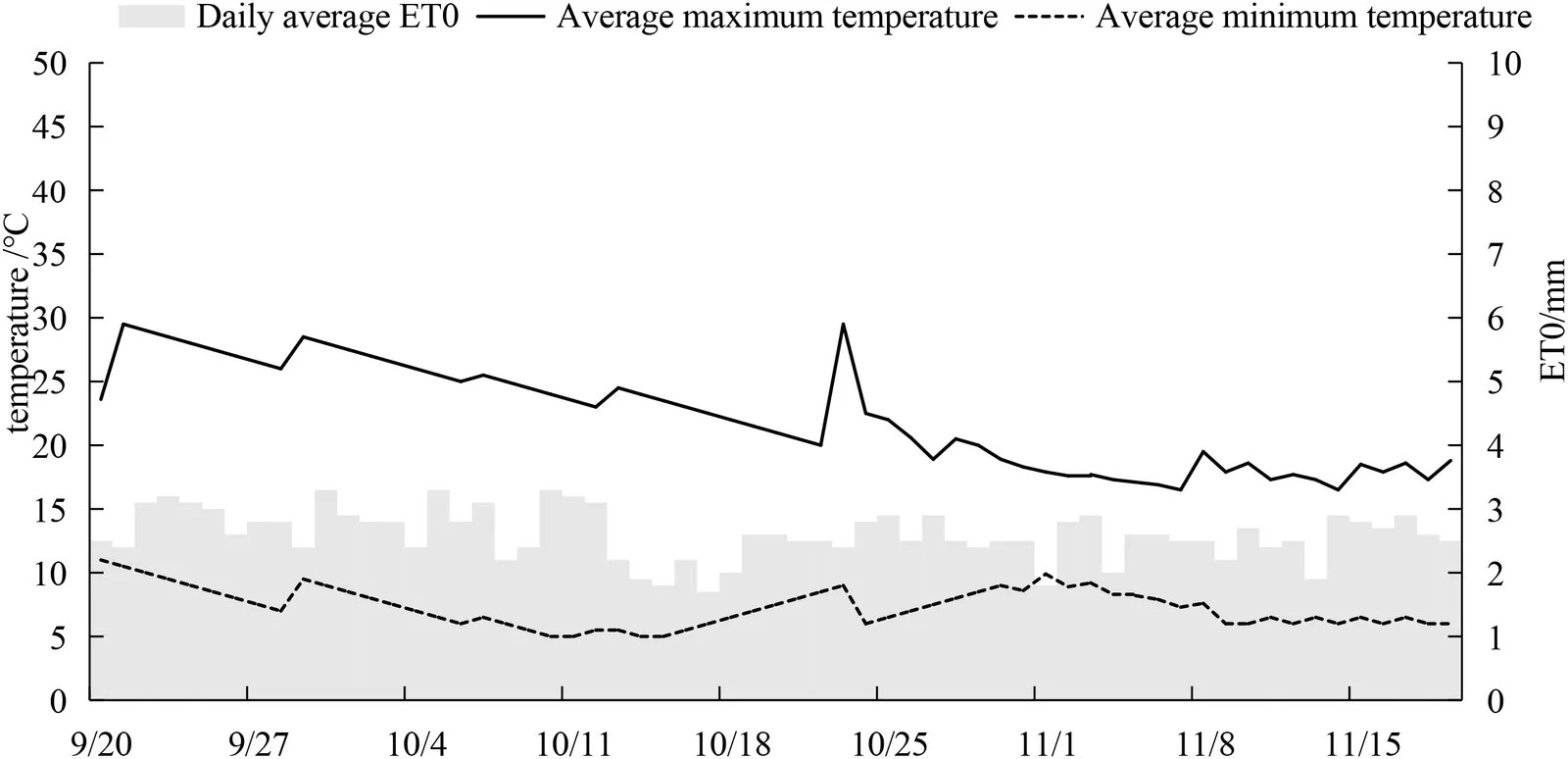
Daily average summer temperature and reference evapotranspiration data of the experimental site.

**Figure 4:**
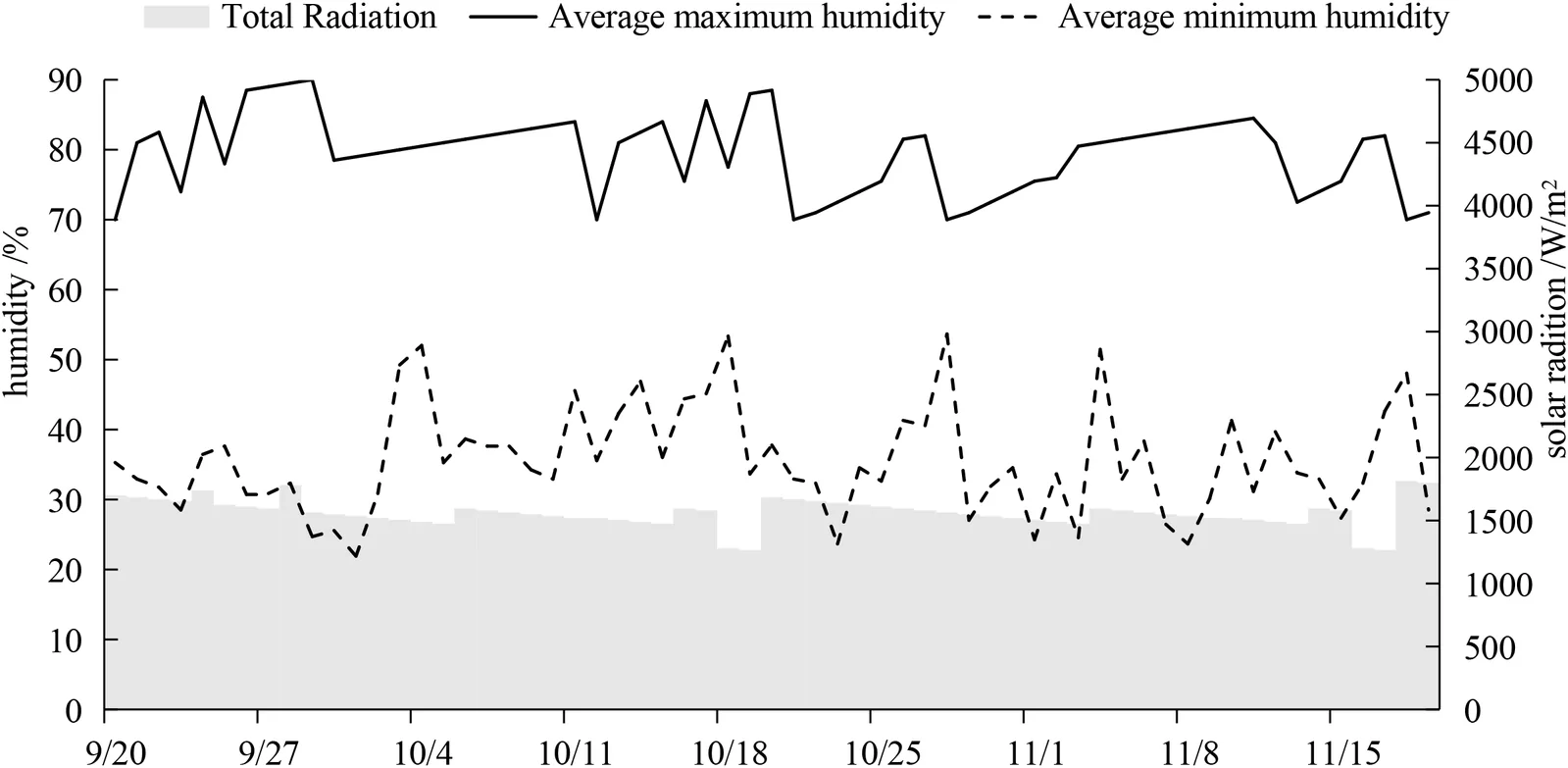
Daily average humidity and solar radiation data of the experimental site in summ.

The experiment took place in Shuixigou Town, Urumqi County, Urumqi region in 2024, located at 87°30′12″E, 43°27′53″N, with an altitude of 1593 meters and situated 40 kilometers away from Urumqi’s urban area. **Figures 1**–**4** illustrate the maximum temperature, minimum temperature, and reference crop evapotranspiration during both summer and winter in the experimental period. **Figure 1** displays the daily average reference evapotranspiration (ET_0_) for summer white radish, ranging from 3.8 to 5.8 mm. The consistently high ET values suggest intense evaporation in the area, attributed to the region’s high temperatures, low humidity, and ample sunshine during summer. In summer, **Figure 2** shows that daily average solar radiation in the greenhouse ranges from 1265 to 1815 W/m^2^. For winter white radish, **Figure 3** indicates that the daily average ET ranges from 1.7 to 3.3 mm, while **Figure 4** shows that daily average solar radiation in the greenhouse during winter varies from 179.75 to 3114.00 W/m^2^. The mechanical composition of soil particle size in the test soil layers of 0–20 cm, 20–40 cm and 40–60 cm in the test area is shown in **Table 1**. The soil texture of the test site is sandy loam. The initial soil nutrient content of the test site is shown in **Table 2**.

**Table 1:** Particle size composition of experimental sites.

Soil depth (cm)	Particle size fraction %		
	Clay particle (<0.002mm)	Silt (0.002∼0.02mm)	Sand (0.02∼2mm)
0-20	33.0	55.9	11.1
20-40	34.7	52	13.3
40-60	33	56.9	10.1

**Table 2:** Initial soil nutrient content at the experimental site.

Crop	Depth	Ammonium nitrogen (mg/kg)	Available potassium (mg/kg)	Available phosphorus (mg/kg)
White radish	0-10cm	5.13	319.905	13.654
	10-20cm	4.92	302.564	13.54
	20-30cm	3.96	298.564	11.881

### 2.2 Experimental design

The study employed the Box-Behnken response surface method to optimize the irrigation and fertilization regimen for greenhouse white radishes. Three factors were considered: nitrogen application rate (N), potassium application rate (K), and irrigation amount (W), each at three levels. Irrigation was based on reference crop evapotranspiration (ET_0_), while nitrogen and potassium rates were determined based on local fertilization standards. The experiment, conducted in both summer and winter, utilized a Box-Behnken design with three center points and a total of 15 treatments. The local standard (W1K1N1) served as the control. Experimental parameters and levels for summer and winter white radishes are detailed in **Tables 3**–**6**. In summer, radishes were irrigated twice weekly and once weekly in winter, with fertilization applied in four installments using urea and potassium oxide as nitrogen and potassium sources, respectively. Each treatment plot measured 32 m² with two ridges and single-row planting. Drip irrigation was employed with a dripper spacing of 30 cm, drip tape spacing of 1 m, and 1.5 m spacing between plots. The growth stages for summer radishes were: seedling (May 20th - May 31st), rosette (June 1st - June 10th), fleshy root expansion (June 11th - June 20th), and harvest (June 21st - July 15th). For winter radishes, stages were: seedling (September 20th - October 10th), rosette (October 11th - November 2nd), fleshy root expansion (November 3rd - November 23rd), and harvest (November 24th - December 15th).

**Table 3:** Experimental factors and level composition of summer radish.

Factor	Level		
	0	1	2
W (irrigation quota)	20	30	40
K (application amount of potassium fertilizer)	100	180	260
N (application amount of nitrogen fertilizer)	140	180	220

**Table 4:** Experimental design of response surface methodology for summer radish.

Processing number	Irrigation quota (mm)	Application amount of potassium fertilizer (kg/hm^2^)	Application amount of nitrogen fertilizer (kg/hm^2^)
1 (W0K1N0)	20	100	180
2 (W2K1N0)	40	100	180
3 (W0K1N2)	20	260	180
4 (W2K1N2)	40	260	180
5 (W0K0N1)	20	180	140
6 (W2K0N1)	40	180	140
7 (W0K2N1)	20	180	220
8 (W2K2N1)	40	180	220
9 (W1K0N0)	30	100	140
10 (W1K0N2)	30	260	140
11 (W1K2N0)	30	100	220
12 (W1K2N2)	30	260	220
13 (W1K1N1)	30	180	180
14 (W1K1N1)	30	180	180
15 (W1K1N1)	30	180	180

**Table 5:** Experimental factors and levels of winter radish.

Factor	Level		
	0	1	2
W (irrigation quota)	15	22.5	30
K (application amount of potassium fertilizer)	100	180	260
N (application amount of nitrogen fertilizer)	140	180	220

**Table 6:** Experimental design of response surface method for winter radish.

Processing number	Irrigation quota (mm)	Application amount of potassium fertilizer (kg/hm^2^)	Application amount of nitrogen fertilizer (kg/hm^2^)
1 (W0K1N0)	15	100	180
2 (W2K1N0)	30	100	180
3 (W0K1N2)	15	260	180
4 (W2K1N2)	30	260	180
5 (W0K0N1)	15	180	140
6 (W2K0N1)	30	180	140
7 (W0K2N1)	15	180	220
8 (W2K2N1)	30	180	220
9 (W1K0N0)	22.5	100	140
10 (W1K0N2)	22.5	260	140
11 (W1K2N0)	22.5	100	220
12 (W1K2N2)	22.5	260	220
13 (W1K1N1)	22.5	180	180
14 (W1K1N1)	22.5	180	180
15 (W1K1N1)	22.5	180	180

### 2.3 Determination indicators and methods

#### 2.3.1 Plant height and stem diameter

Five plants displaying uniform growth were chosen and labeled in every plot. Plant height was measured from the base to the tallest point of the aerial portion using a tape measure accurate to.1 cm, while stem diameter was measured with a vernier caliper accurate to.02 mm at the seedling, rosette, fleshy root expansion, and harvest stages of white radish plants.

#### 2.3.2 Dry matter mass

During various growth stages of white radishes, including seedling, rosette, fleshy root expansion, and harvest stages, three uniform plants were chosen from each plot for analysis. Dry matter weights of plant organs were determined using the whole-plant harvest technique. Samples were dried at 65°C until reaching a constant weight and then weighed with a precision of.01 g. The root-to-shoot ratio was calculated using Equation 1.

(1) $$\frac{R}{S}=\frac{M_{1}}{M_{2}}$$

M1: The dry mass of the root, with the unit of g.

M2 denotes the dry weight of the aerial components (such as stems, leaves, etc.), measured in grams (g).

#### 2.3.3 Irrigation water use efficiency

Following the completion of the entire growth period of white radishes, the irrigation water use efficiency for each treatment was assessed using the formula outlined in Equation 2.

(2) $$IWUE=\frac{Y}{I}$$

IWUE: Irrigation water use efficiency, with the unit of kg/(hm²·mm).

Y represents the yield of mature greenhouse-grown white radishes, measured in kg/hm².

Irrigation quota for the entire growth period of greenhouse radish is expressed in millimeters (mm).

(3) Crop yield and fruit shape index

At the white radish harvest stage, the fresh weight of individual white radishes in each plot was measured using a platform scale with a precision of 1 g. The total yield was then determined and converted to kg/hm². To assess fruit shape, three fruits were randomly sampled from each treatment plot at various growth stages (seedling, rosette, fleshy swelling, and harvest stages) of greenhouse white radishes. The transverse and longitudinal diameters of the fruits were measured with a vernier caliper to calculate the fruit shape index.

#### 2.3.4 Fruit quality

During the maturity stage, three plants were randomly chosen from each plot for the assessment of vitamin C and soluble sugars ^[21]^. The anthrone colorimetric method was utilized for quantifying soluble sugars, while the molybdenum blue colorimetric method was employed for determining the vitamin C content in the fruits ^[21]^.

#### 2.3.5 Partial factor productivity of fertilizer

At the conclusion of the entire growth period of white radishes, the partial factor productivity of fertilizers for each treatment was assessed as depicted in Equation 3.

(3) $$PFP=\frac{Y}{F}$$

Y represents the yield in the fertilized area, denoting the crop yield obtained from experimental plots where nitrogen and potassium fertilizers are concurrently applied, measured in kg/hm².

Fertilization amount (referred to as the quantity of nitrogen or potassium fertilizer applied) is measured in kg/hm².

#### 2.3.6 Meteorological data

Data on air humidity, temperature, solar radiation, and reference crop evapotranspiration were obtained through measurements and calculations conducted by a compact weather station.

### 2.4 Data processing

The Design-Expert 11 software was used for experimental design and multivariate statistical regression analysis. All data were processed by Microsoft Excel 2013 and analyzed using the SPSS 20.0 statistical software. The least significant difference test (LSD) was employed to determine the significance of differences among treatments (p < 0.05). All figures were plotted using the Origin 2022 and Microsoft Excel 2013 software.

## 3 Results and analysis

### 3.1 Effects of water-fertilizer coupling on physiological indicators of greenhouse radishes

#### 3.1.1 Summer

Analysis of **Table 7** shows that the maximum value of greenhouse summer radish plant height appeared in the W1K1N1 treatment (28.5 cm), which was 0.96% higher than the control; the maximum value of stem thickness appeared in the W1K0N0 treatment (42.6 mm), which was 9.60%; the maximum value of root-crown ratio appeared in W0K0N1 treatment (1.95), upgraded by 72.57% compared with the control; the maximum value of leaf area index appeared in W0K2N1 treatment (0.334), upgraded by The maximum value of leaf area index appeared in W0K2N1 treatment (0.334), upgrading 25.05% compared with the control. Comprehensively, W2K1N2 was the best in biomass accumulation, while W1K0N0 and W0K0N1 were the best in stem thickness and root development, respectively. significant advantages in stem thickness and root development, respectively. Plant height tended to increase and then decrease with the increase of irrigation (W0→W2), such as W1K1N1 (28.23 cm) was 11.1% higher than W0K1N0 (25.4 cm), but excessive irrigation (W2) led to a decrease in plant height (25.9 cm for W2K1N2). Drought treatment (W0) significantly increased root-crown ratio, consistent with the stress response of resource allocation to the root system ^[30]^, but suppressed aboveground biomass (45.32 g dry matter mass for W0K1N0 vs. 59.82 g for W2K1N2). Stem thickness and leaf area index showed an increasing and then decreasing trend with increasing nitrogen application (N0→N2), e.g., stem thickness of W1K1N1 was 38.87 mm in the medium nitrogen (N1) treatment, whereas it was reduced to 37.6 mm in the high-nitrogen (N2) treatment of W1K0N2, which was related to the inhibition of cell elongation by nitrogen excess ^[22]^. High potassium (K2) significantly elevated the leaf area index to 0.334 under drought conditions (W0K2N1), but potassium deficiency (K0) induced an elevated root-crown ratio (up to 1.95 in W0K0N1), reflecting that potassium deficiency promotes compensatory root growth^[23]^. In conclusion, it can be concluded that W2K1N2 is the optimum biomass production combination, while W0K0N1 and W1K0N0 are suitable for root and stem trait improvement, respectively.

**Table 7:** Physiological indicators of mature white radish.

Processing number	Plant height(cm)		Stem diameter(mm)		Root-cap ratio		Leaf area index		The mass of dry matter inthe above-ground part(g)	
	Summer	Winter	Summer	Winter	Summer	Winter	Summer	Winter	Summer	Winter
1	25.4 ± 4.6a	22.5 ± 1.1b	33.3 ± 8.58b	33.48 ± 4.9b	1.03 ± 1.26a	1.18 ± 0.12c	0.313 ± 0.01a	0.442 ± 0.21ab	45.32 ± 4.23def	35.1 ± 1.2de
2	24.8 ± 4.5a	31.1 ± 1.8b	33.1 ± 8.1b	34.05 ± 5.6ab	1.08 ± 1.28a	1.24 ± 0.09c	0.307 ± 0.02a	0.443 ± 0.43ab	22.42 ± 2.21i	12.2 ± 4.8h
3	26.2 ± 3.8a	26.0 ± 6.5b	31.1 ± 6.2b	33.7 ± 5.1ab	1.04 ± 1.25a	1.21 ± 0.06c	0.299 ± 0.03a	0.424 ± 0.42bc	51.42 ± 8.72cde	41.2 ± 1.8c
4	25.9 ± 2.6a	24.5 ± 6.2b	31.8 ± 6.3b	33.48 ± 5.1ab	1.19 ± 1.35a	1.33 ± 0.17bc	0.314 ± 0.08a	0.434 ± 0.33ab	59.82 ± 6.3a	49.6 ± 2.4a
5	27.0 ± 2.35a	27.0 ± 7.1b	32.6 ± 7.2b	33.58 ± 5.3ab	1.95 ± 2.03a	2.22 ± 0.24a	0.328 ± 0.04a	0.436 ± 0.33ab	44.12 ± 3.2ef	33.9 ± 1.5de
6	26.5 ± 3.2a	29.1 ± 6.9b	33.5 ± 6.3b	34.61 ± 5.5ab	1.05 ± 1.29a	1.28 ± 0.10c	0.333 ± 0.01a	0.435 ± 0.33ab	30.42 ± 6.1h	30.2 ± 1.2ef
7	27.5 ± 1.6a	32.5 ± 5.5b	35.0 ± 3.7b	38.93 ± 4.5ab	1.88 ± 1.98a	2.08 ± 0.19bc	0.334 ± 0.02a	0.331 ± 0.33c	52.33 ± 6.0bcd	42.1 ± 2.1bc
8	27.2 ± 2.8a	30.5 ± 6.5b	29.23 ± 2.3bc	33.59 ± 4.5b	1.18 ± 1.33a	1.36 ± 0.18bc	0.323 ± 0.04a	0.445 ± 0.41ab	33.92 ± 5.9gh	23.7 ± 3.5g
9	25.6 ± 3.4a	23.5 ± 6.3b	42.6 ± 10.3a	34.15 ± 8.4a	1.30 ± 1.36a	1.52 ± 0.19bc	0.321 ± 0.03a	0.419 ± 0.42bc	59.02 ± 4.3ab	48.8 ± 2.2a
10	26.8 ± 1.9a	27 ± 3.9b	37.6 ± 9.5ab	33.4 ± 6.1ab	1.94 ± 2.02a	2.18 ± 0.23a	0.331 ± 0.03a	0.313 ± 0.31c	57.02 ± 3.6abc	46.8 ± 2.6ab
11	27.3 ± 2.65a	22.5 ± 4.4b	36.9 ± 3.8ab	34.72 ± 7.4ab	1.57 ± 1.77a	1.82 ± 0.21abc	0.322 ± 0.01a	0.426 ± 0.44bc	38.72 ± 7.1fg	28.5 ± 1.3fg
12	27.1 ± 3.9a	27.5 ± 4.1b	37.6 ± 10.31ab	33.4 ± 7.2ab	0.94 ± 1.65a	1.16 ± 0.21abc	0.308 ± 0.02a	0.428 ± 0.42bcd	33.92 ± 6.2gh	23.7 ± 3.1g
13	28.0 ± 3.4a	30.5 ± 6.5a	38.8 ± 10.35ab	34.41 ± 8.2ab	1.13 ± 1.31a	1.29 ± 0.16bc	0.327 ± 0.02a	0.429 ± 0.35bcd	47.02 ± 6.7de	36.8 ± 1.6cd
14	28.2 ± 3.3a	30.5 ± 6.5a	38.8 ± 10.15ab	34.42 ± ab	1.13 ± 1.32a	1.31 ± 0.16bc	0.321 ± 0.03a	0.429 ± 0.31bc	48.22 ± 7.1de	38.7 ± 1.9cd
15	28.5 ± 2.6a	30.6 ± 6.4a	38.9 ± 9.85ab	34.5 ± ab1	1.12 ± 1.30a	1.30 ± 0.16bc	0.319 ± 0.02a	0.429 ± 0.43bc	48.22 ± 6.8de	38.9 ± 1.8cd

Means within the same column followed by different lowercase letters indicate significant differences among treatments at the 5% level.

#### 3.1.2 Winter

According to the analysis in **Table 7**, the maximum value of winter greenhouse white radish plant height appeared in W0K2N1 treatment (32.5 cm), which was elevated by 8.22% compared with the control group. The maximum value of stem thickness appeared in W0K2N1 treatment (38.93 mm), which was 13.00% higher than the control group, and the maximum value of root-crown ratio appeared in W0K0N1 treatment (2.22), which was 70.77% higher than the control group. The maximum value of leaf area index appeared in W2K2N1 treatment (0.445), which was 3.73% higher than the control. The maximum value of dry matter mass of above ground portion appeared in W2K1N2 treatment (49.6 g), which was enhanced by 30.07 per cent over the control. Plant height was 6.56% higher in W0K2N1 (32.5 cm) compared to W2K2N1 (30.5 cm), indicating that moderate water deficit (W0) in winter greenhouse was more favorable for plant height development, which was different from summer response pattern. Excessive watering (W2) led to a reduction in aboveground biomass of W2K1N0 to 12.2 g, 65.24% lower than that of W0K1N0 (35.1 g), suggesting that the osmotic water absorption efficiency of the root system was more sensitive to water excess in winter. High potassium (K2) significantly enhanced stem thickness to 38.93 mm in drought conditions (W0K2N1), which was 15.93% higher than that of W0K0N1 (33.58 mm), corroborating that potassium ions enhance the mechanical strength of the cell wall by regulating osmotic pressure. High nitrogen (N2) significantly enhanced aboveground biomass to 49.6 g in W2K1N2, which was 27.84% higher than that of medium nitrogen (N1) treatment W2K1N1 (mean 38.8 g), but excess nitrogen led to a reduction in stem thickness to 33.4 mm in W1K0N2, which was 2.25% less than that of W1K0N0 (34.15 mm), consistent with the biological inhibition of cytokinin synthesis by excessive nitrogen mechanism. The optimal biomass accumulation combination for greenhouse white radish in winter was W2K1N2, while W0K2N1 and W0K0N1 were suitable for stem trait improvement and drought tolerant cultivation, respectively. Compared with summer, the treatment corresponding to the maximum plant height in winter shifted from W1K1N1 (28.5 cm in summer) to W0K2N1 (32.5 cm in winter), with an increase of 14.04%, suggesting that the reciprocal effect of moderate water stress (W0) and high potassium (K2) was more significant in low-temperature environments. Root-crown ratio elevation was lower in winter (70.77%) than in summer (72.57%), reflecting that seasonal temperature differences affected the intensity of root stress response.

### 3.2 Effects of water and fertilizer coupling on soil nutrients at different depths

The nutrient distribution characteristics of summer greenhouse white radish soil are shown in **Table 8**. Available potassium and ammonium nitrogen show significant surface aggregation effect (p<0.05). Available potassium content in 10 cm soil layer (mean 102.17 mg/kg) was significantly higher than that in 20 cm (84.41 mg/kg) and 30 cm (73.73 mg/kg), and the vertical attenuation rate was 27.9%. W2K2N1 treatment (treatment 4) reached the peak of 206.97 mg/kg available potassium at 10 cm depth, which was 148.5% higher than that of W1K1N1 (treatment 13–15 mean 83.29 mg/kg), indicating that high irrigation rate (W2) and high potassium (K2) promoted potassium accumulation in surface layer. Ammonium nitrogen surface aggregation effect is more significant, 10 cm layer mean (109.74 mg/kg) vs. 20 cm (85.64 mg/kg) and 30 cm (76.30 mg/kg) increased by 28.2% and 43.9% respectively, W1K2N0 treatment (treatment 11) reached a peak of 172.51 mg/kg at 10 cm, which was 75.9% higher than that of control (treatment 13–15 mean 98.03 mg/kg), indicating that low irrigation (W1) and high potassium (K2) combination enhanced nitrogen retention in surface layer by inhibiting ammonium fixation.

**Table 8:** Soil Nutrients at different depths during the maturity stage of white radish in summer greenhouse.

Treatment	10cm Quick acting potassium (mg/kg)	10cm Ammonium nitrogen (mg/kg)	20cm Quick acting potassium(mg/kg)	10cm Ammonium nitrogen (mg/kg)	20cm Quick acting potassium (mg/kg)	30cm Ammonium nitrogen (mg/kg)
1	93.07 ± 3.14bc	103.17 ± 3.14bc	90.337 ± 29.15e	55.37 ± 29.15e	112.01 ± 41.37ab	112.08 ± 41.3ab
2	99.45 ± 1.55bc	99.45 ± 1.55bc	54.53 ± 5.25e	54.51 ± 5.25e	79.57 ± 3.42bc	79.77 ± 3.42bc
3	108.07 ± 51.21ab	108.07 ± 51.2ab	50.513 ± 58.24e	53.51 ± 58.24e	79.57 ± 3.42bc	75.77 ± 3.42bc
4	206.97 ± 52.29a	191.8 ± 52.29a	64.82 ± 19.06d	68.22 ± 19.06d	58.87 ± 2.01e	59.65 ± 2.01e
5	64.82 ± 19.06e	64.82 ± 19.06e	71.19 ± 8.36bcd	71.89 ± 8.36bcd	61.38 ± 6.29e	63.75 ± 6.29e
6	58.865 ± 2.01e	59.61 ± 2.01e	83.30 ± 38.51bc	83.02 ± 38.51bc	83.13 ± 38.45bcd	83.30 ± 38.45bcd
7	58.865 ± 2.01e	58.86 ± 2.01e	83.30 ± 34.51bc	83.02 ± 34.51bc	85.30 ± 38.45bcd	87.30 ± 38.45bcd
8	127.58 ± 81.98ab	127.58 ± 81.9ab	124.91 ± 61.69ab	129.06 ± 61.6ab	69.12 ± 19.14e	63.12 ± 19.14e
9	84.53 ± 1.18c	82.53 ± 1.18c	90.54 ± 7.24bc	90.42 ± 7.24bc	104.98 ± 17.47ab	119.75 ± 17.4ab
10	84.375 ± 2.50c	83.75 ± 2.50c	108.81 ± 20.87ab	118.08 ± 20.8ab	79.71 ± 25.40bcd	79.10 ± 25.40bcd
11	89.94 ± 26.07c	84.44 ± 26.07c	162.05 ± 49.45a	172.51 ± 49.45a	90.26 ± 34.38e	92.57 ± 34.38e
12	69.81 ± 33.51d	70.17 ± 33.51d	99.58 ± 17.25ab	99.80 ± 17.25ab	112.85 ± 25.95ab	112.50 ± 25.95ab
13	82.29 ± 29.20cd	83.99 ± 29.20cd	95.90 ± 34.61ab	95.03 ± 34.61ab	82.30 ± 29.20cd	82.99 ± 29.20cd
14	82.31 ± 29.10cd	83.20 ± 29.15cd	95.92 ± 34.50ab	99.20 ± 34.55ab	82.31 ± 29.10cd	83.20 ± 29.15cd
15	82.28 ± 29.30cd	82.78 ± 29.25cd	95.88 ± 34.72ab	98.86 ± 34.67ab	82.28 ± 29.30cd	82.78 ± 29.25cd

Means within the same column followed by different lowercase letters indicate significant differences among treatments at the 5% level.

The analysis of interaction between water and fertilizer showed that excessive irrigation (W2) resulted in the aggravation of available K leaching, for example, treatment 8 (W2K1N0) reduced available K (69.12 mg/kg) at 30 cm by 45.8% compared with 10 cm (127.58 mg/kg). N 2 treatment was outstanding at 20 cm, and available K in treatment 10 (W1K1N2) was 118.08 mg/kg, which was 24.4% higher than that of CK. It is worth noting that the combination of K2 and W1 (treatment 11) reached 162.05 mg/kg of available potassium at 20 cm, forming a subsurface enrichment peak, revealing the vertical migration characteristics of potassium fertilizer under moderate moisture.

The data in **Table 9** show that soil ammonium nitrogen and available potassium in the mature stage of white radish in winter greenhouse show significant enrichment characteristics in the surface layer. The mean ammonium concentration in 10 cm soil layer (113.24 mg/kg) was 33.8% and 49.3% higher than that in 20 cm soil layer (84.62 mg/kg) and 30 cm soil layer (75.84 mg/kg), respectively, with a vertical decay rate of 32.9%. The ammonium content in 10 cm of treatment 4 reached 206.99 ± 52.29 mg/kg, which was significantly higher than other treatments (P<0.05) and 69.8% higher than that of the control treatment 13 (121.85 ± 13.34 mg/kg), indicating that high nitrogen input may inhibit nitrogen downward migration. Notably, treatment 11 showed an abnormal peak of ammonium (162.05 ± 49.45 mg/kg) at 20 cm soil layer, which might be related to local uneven fertilization or heterogeneous root distribution.

**Table 9:** Soil nutrients at different depths during the maturity stage of winter greenhouse white radish.

Treatment	10cm Quick acting potassium (mg/kg)	10cm Ammonium nitrogen (mg/kg)	20cm Quick acting potassium (mg/kg)	10cm Ammonium nitrogen (mg/kg)	20cm Quick acting potassium (mg/kg)	30cm Ammonium nitrogen (mg/kg)
1	196.49 ± 8.25bc	93.02 ± 3.14bc	179.32 ± 2.76d	64.30 ± 75.05i	185.98 ± 5.21cd	58.91 ± 68.76e
2	271.04 ± 146.7ab	99.45 ± 1.55bc	181.37 ± 2.31d	54.51 ± 5.25e	220.21 ± 9.38c	79.57 ± 3.42bc
3	237.71 ± 105.3ab	108.08 ± 51.21ab	196.33 ± 51.98d	50.51 ± 58.24e	223.88 ± 10.82ab	79.57 ± 3.42bc
4	223.91 ± 11.12a	206.99 ± 52.29a	240.33 ± 14.43b	64.82 ± 19.06d	266.67 ± 23.09a	58.86 ± 2.01e
5	215.21 ± 67.69d	103.86 ± 47.54f	224.37 ± 0.01ab	71.14 ± 37.88i	194.74 ± 46.79cd	38.22 ± 7.52g
6	221.67 ± 24.62ab	101.76 ± 16.32f	220.15 ± 8.91bc	72.82 ± 48.33i	195.29 ± 6.17cd	66.02 ± 38.02e
7	204.86 ± 16.20bc	58.86 ± 2.01e	193.42 ± 5.777cd	83.30 ± 34.51bc	192.08 ± 11.54cd	83.13 ± 38.45bcd
8	190.29 ± 57.66bc	127.59 ± 81.98ab	214.54 ± 115.85bc	124.91 ± 61.69ab	201.16 ± 6.89e	69.31 ± 19.14e
9	171.63 ± 4.333d	84.35 ± 1.18c	202.59 ± 28.56bc	80.43 ± 19.95g	227.04 ± 11.5ab	65.54 ± 54.98e
10	183.44 ± 43.88d	84.37 ± 2.50c	198.98 ± 5.83c	83.92 ± 5.666f	145.45 ± 10.24e	82.99 ± 47.84cd
11	251.829 ± 4.60ab	89.94 ± 26.07c	275.745 ± 102.37a	162.05 ± 49.45a	191.15 ± 6.89bc	90.25 ± 34.38e
12	201.77 ± 18.67bc	69.82 ± 33.51d	266.74 ± 109.3a	99.58 ± 17.25ab	208.96 ± 57.84bc	112.85 ± 25.95ab
13	219.57 ± 61.90ab	121.85 ± 13.34a	220.99 ± 67.21c	104.09 ± 66.78bc	234.93 ± 101.15ab	89.26 ± 27.35cd
14	219.60 ± 62.00ab	121.90 ± 13.30a	221.02 ± 67.30c	104.12 ± 66.70bc	23.50 ± 101.20ab	89.30 ± 27.30cd
15	219.55 ± 61.80ab	121.80 ± 13.38a	220.98 ± 67.10c	104.06 ± 66.86bc	234.85 ± 101.10ab	89.22 ± 27.40cd

Means within the same column followed by different lowercase letters indicate significant differences among treatments at the 5% level.

The surface aggregation effect of available potassium was more significant, with the mean value of 10 cm soil layer (213.57 mg/kg) being 1.5% and 12.3% higher than that of 20 cm (210.44 mg/kg) and 30 cm (190.28 mg/kg), respectively. The available potassium content at 20 cm in treatment 11 reached 275.75 ± 102.37 mg/kg, the highest value in the whole profile, which was 24.8% higher than that in the control treatment 13 (221.02 ± 67.30 mg/kg), suggesting that K2 fertilization level inhibited potassium migration to deeper layers through ion antagonism. In particular, available potassium in the 30 cm soil layer of treatment 14 suddenly decreased to 23.50 ± 101.20 mg/kg, with a large data dispersion (CV = 430.6%), which might be due to sampling error or local leaching aggravation. The coefficients of variation among treatments were generally high (for example, the CV of available potassium in treatment 2 was 54.1%), reflecting the significant effect of greenhouse microenvironment heterogeneity on nutrient distribution. Our results showed that nitrogen reduction combined with high potassium application (e.g., treatment 11) could enhance surface potassium retention, but the risk of salinization caused by excessive accumulation should be paid attention to.

### 3.3 Effect of water and fertilizer coupling on yield and quality

#### 3.3.1 Irrigation water use efficiency and fertilizer partial productivity

**Figure 5:**
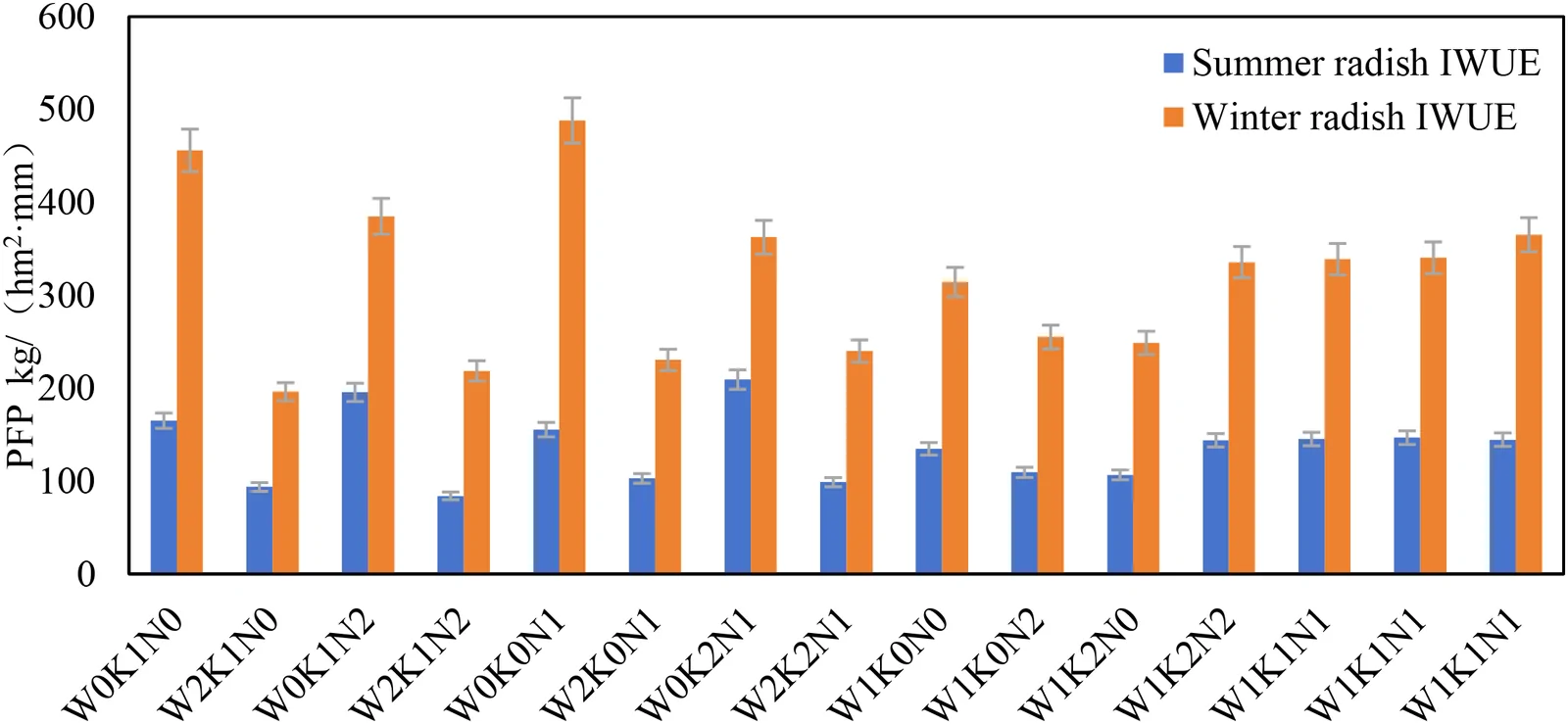
The utilization rate of irrigation water for each treatment of greenhouse radishes in summer and winter.

**Figure 5** illustrates notable seasonal variations in irrigation water use efficiency (IWUE) for greenhouse white radishes under different water and fertilizer regimens. During winter cultivation, the WKN1 treatment combination yielded the highest IWUE at 488.19 kg/(hm²·mm), contrasting with the lowest value of 93.71 kg/(hm²·mm) observed with the W2K1N treatment in summer, reflecting a substantial seasonal disparity of 133.47%. Notably, in summer under W moisture conditions, a rise in nitrogen fertilizer application significantly boosted IWUE. Specifically, increasing nitrogen from N to N1 elevated IWUE from 165.08 kg/(hm²·mm) to 209.27 kg/(hm²·mm). Conversely, in winter under W conditions, escalating nitrogen application led to a marked IWUE decline. Transitioning from N1 to N2 treatment reduced IWUE from 488.19 kg/(hm²·mm) to 362.59 kg/(hm²·mm) (P<0.01). This variation likely stems from seasonal environmental dynamics, where the summer’s high temperatures and humidity escalate soil moisture evaporation, while the winter’s cold and arid conditions impede evaporation processes.

**Figure 6:**
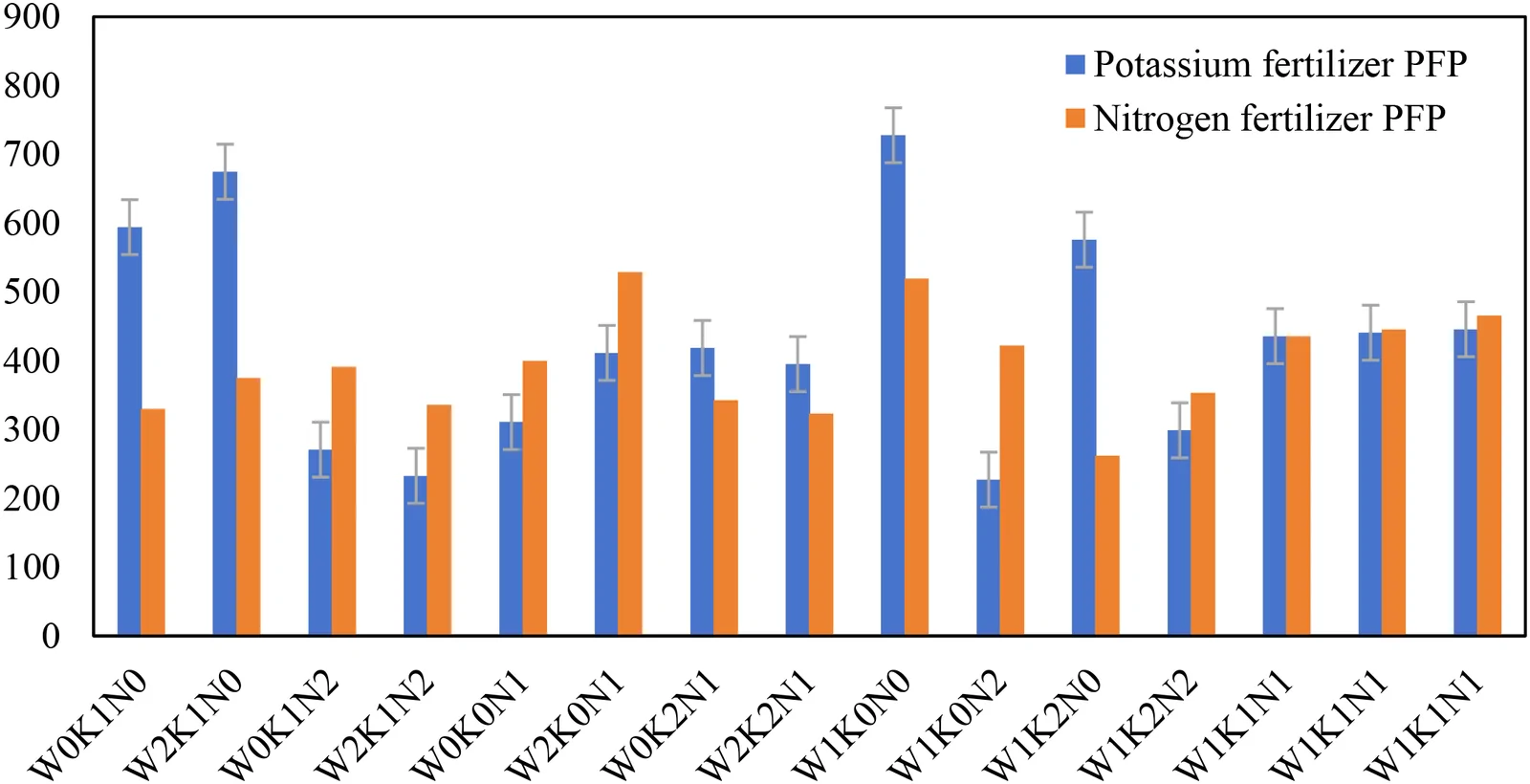
The productivity of greenhouse white radishes treated with various fertilizers in summer.

**Figure 7:**
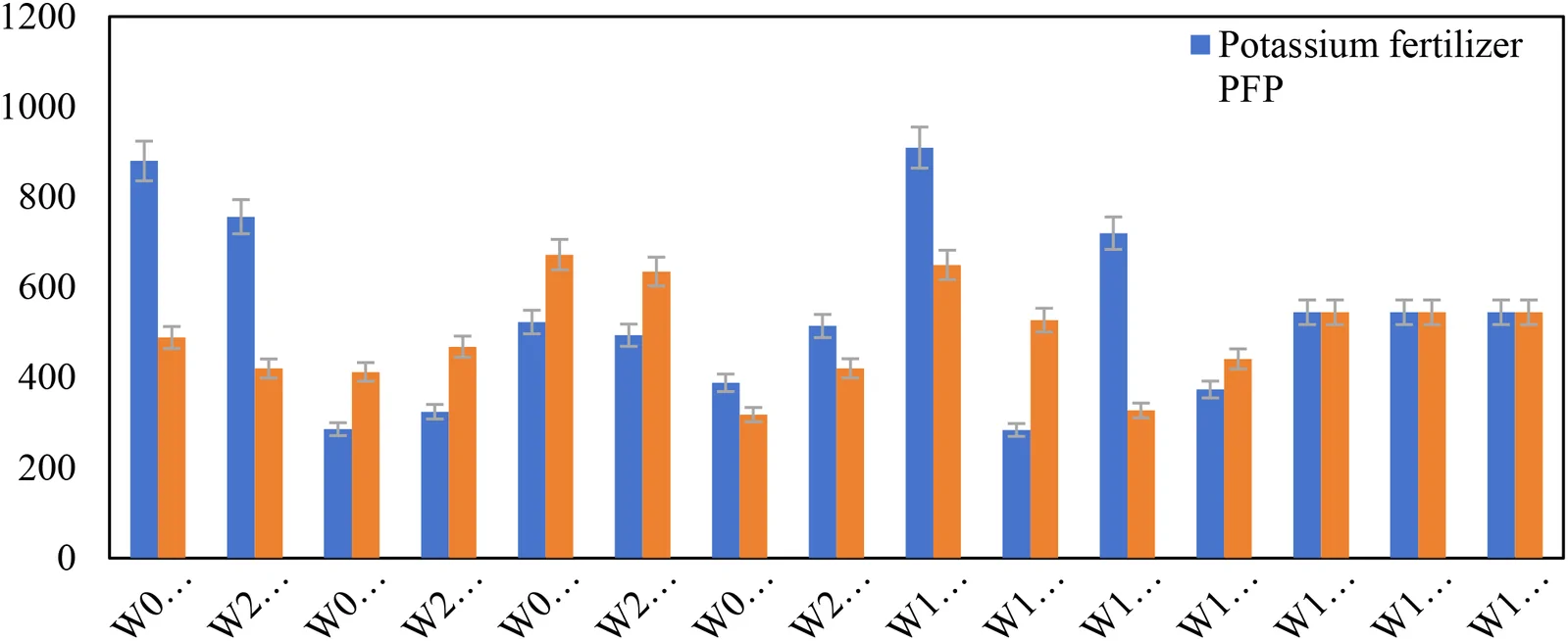
The productivity of greenhouse white radishes treated with various fertilizers in winter.

**Figure 8:**
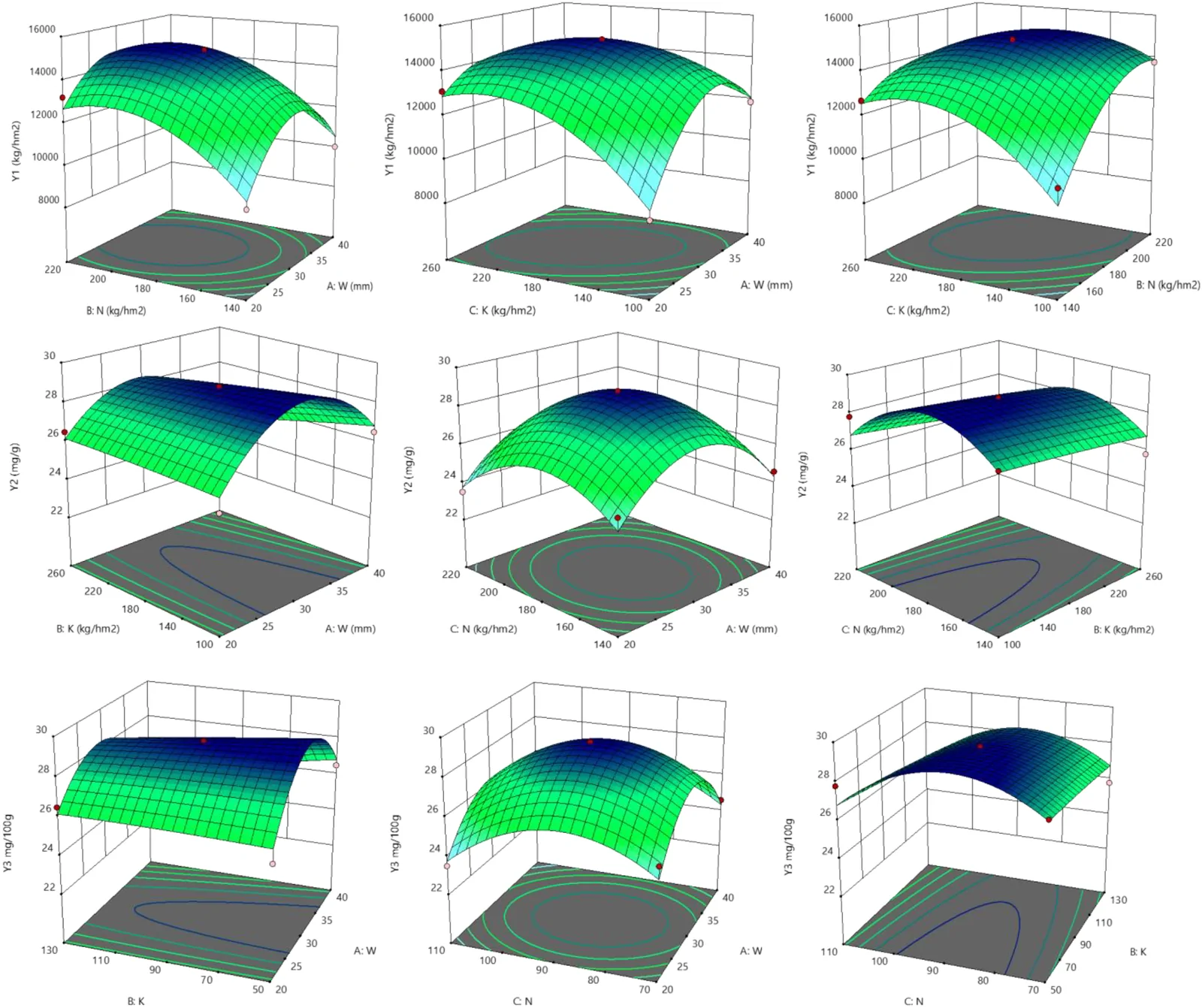
Effects of pairwise interactions of various factors on greenhouse soluble sugar content and vitamin C content.

**Figure 9:**
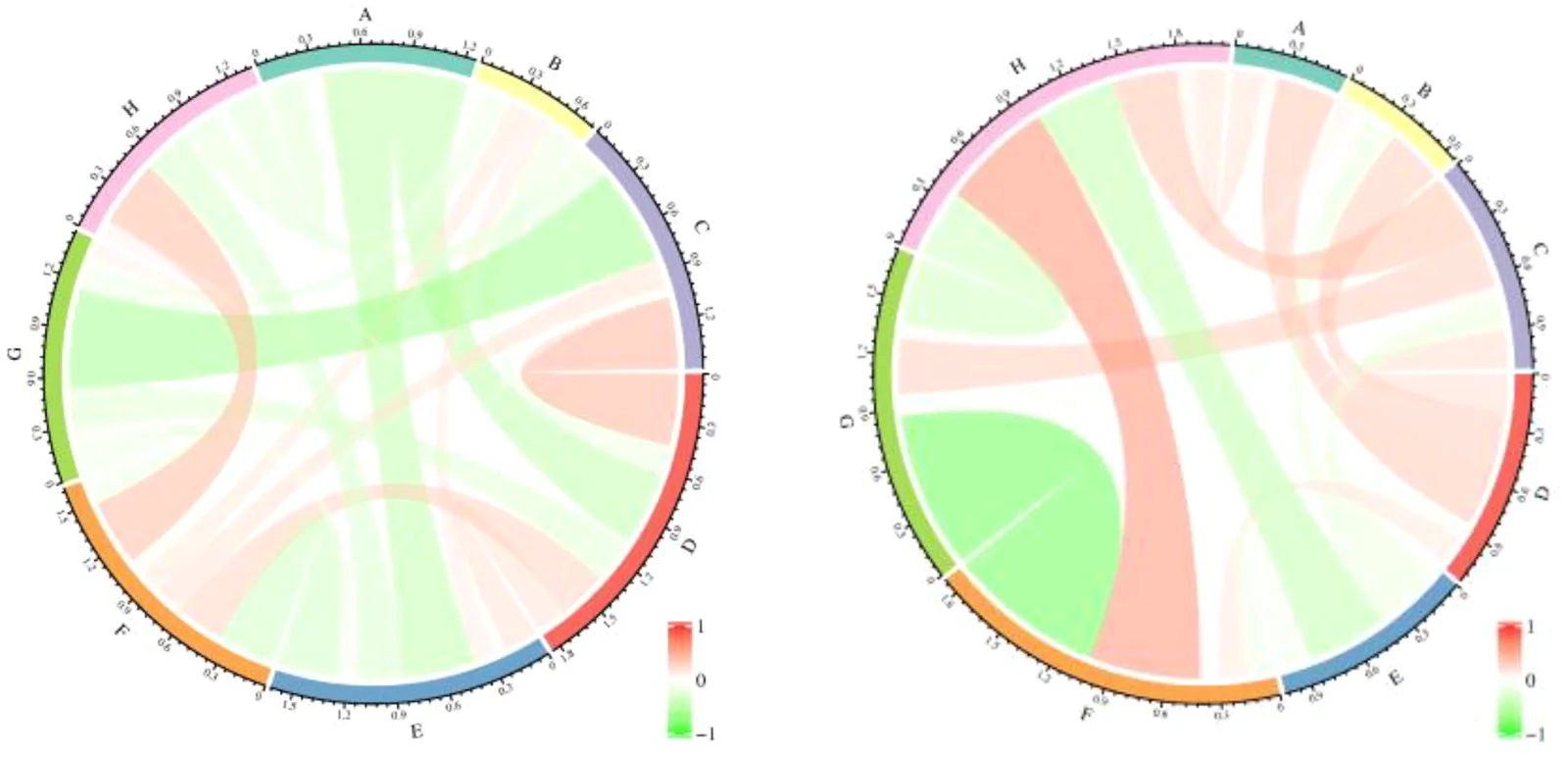
Network graph of correlation coefficients between yield and quality of greenhouse white radishes in summer and winter. [Here, **(A–H)** respectively represent the irrigation quota, nitrogen fertilizer application rate, potassium fertilizer application rate, soluble sugar, vitamin C, fruit volume, fruit shape index, yield, single fruit weight, and yield].

**Figure 10:**
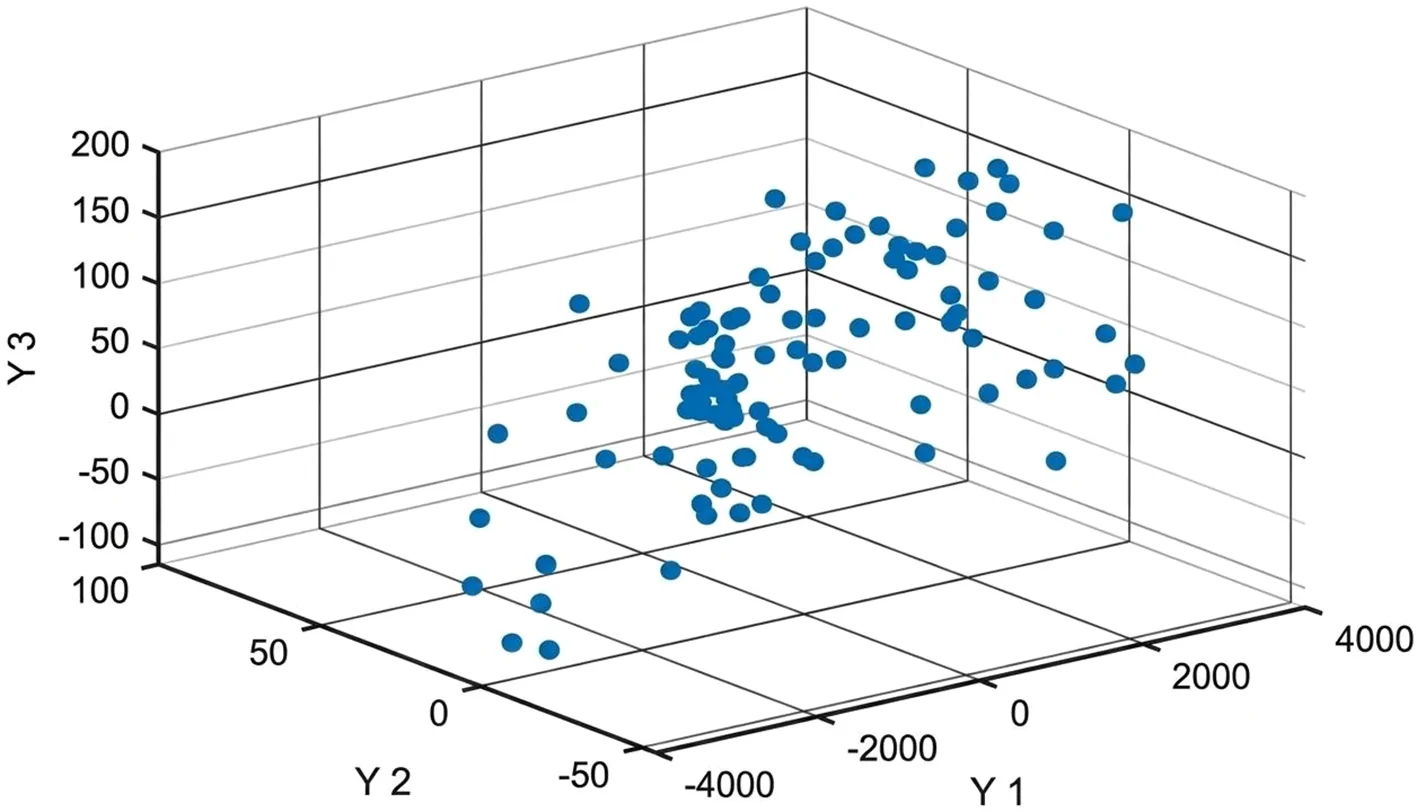
Three dimensional pareto frontier diagram.

It can be known from the data in **Figures 6**, **7** and **Table 8** that the seasonal difference in fertilizer partial productivity (PFP) of greenhouse radishes is significant. As shown in **Figure 8**, the p value of the vitamin C content response surface model was 0.0456 (p < 0.05), indicating that the overall fitting effect of the model reached a significant level. Based on the statistical analysis results of **Figure 9**, there were significant seasonal correlation characteristics between yield and quality indicators of greenhouse radish. The distribution is shown in **Figure 10**. It is generally higher in winter than in summer and is regulated by the coupling effect of water and fertilizer. The average PFP of nitrogen fertilizer in winter (477.6 kg/kg) was 24.5% higher than that in summer (383.7 kg/kg). Among them, the W0K0N1 treatment had the greatest increase, and its PFP of nitrogen fertilizer in winter (672.65 kg/kg) was 68.3% higher than that in summer (399.67 kg/kg) (p<0.05). The seasonal difference of potassium fertilizer PFP is more significant. The average value in winter (534.8 kg/kg) is 33.8% higher than that in summer (399.6 kg/kg). Especially in the W1K0N0 treatment as a typical example, the PFP of potassium fertilizer in winter (909.71 kg/kg) is 25.0% higher than that in summer (727.77 kg/kg). Under the condition of low irrigation volume (W0), the PFP of potassium fertilizer in winter increased by 41.2% compared with that in summer (for example, in the W0K1N0 treatment, it rose from 594.29 to 880 kg/kg), while in the high irrigation volume (W2) treatment, due to the weakened leaching in winter, The increase in PFP of potassium fertilizer narrowed to 12.4% (for example, in the W2K2N1 treatment, it rose from 395.17 to 514.29 kg/kg).High nitrogen application (N2) in summer significantly inhibited PFP. The PFP of nitrogen fertilizer in the W0K1N2 treatment (391.11 kg/kg) was 5.5% lower than that in the same treatment in winter (412.70 kg/kg), which might be related to the acceleration of ammonium nitrogen volatilization by high temperature. The low-temperature environment in winter enhances nutrient efficiency through a dual mechanism: On the one hand, it inhibits the activity of soil microorganisms, reducing the mineralization rate of ammonium nitrogen by 28%-35% and minimizing the loss of ineffective nitrogen; On the other hand, it reduces the fixation intensity of potassium ions by clay minerals, increasing the proportion of available potassium in the root zone by 19%-26%. It is worth noting that the nitrogen fertilizer PFP (420.32 kg/kg) of the W2K1N0 treatment in winter was still significantly lower than the winter average, indicating that excessive irrigation (W2) would lead to intensified longitudinal migration of nutrients and weaken the seasonal gain effect. Studies show that optimizing the ratio of water and fertilizer in winter and summer can synergistically improve the efficiency of resource utilization, but the risk of excessive fertilization induced by high PFP in winter needs to be avoided.

### 3.4 The influence of water and fertilizer coupling on the yield and quality of greenhouse radishes

#### 3.4.1 Seasonal changes in yield and quality of greenhouse radish

According to **Table 10**, the yield of greenhouse white radish in winter is significantly higher than that in summer, with an average increase of 24.3% (p<0.05). The winter yield of W2K1N2 treatment reached 99.94 t/hm ², an increase of 7.96% compared to the mean of W1K1N1 (92.58 t/hm²), which is the highest value in the entire treatment. The summer quality indicators showed a synergistic optimization feature, with treatment W0K2N1 having significantly lower soluble sugars (2.0 mg/g) and vitamin C (23.50 mg/100g) compared to other treatments. However, its summer yield (80.34 t/hm²) increased by 2.4% compared to W1K1N1 (78.45 t/hm²). The fruit volume generally increases in winter, with treatment W0K2N1 reaching 1519.6 cm ³, an increase of 47.8% compared to summer (1028.1 cm³), but accompanied by a decrease in fruit shape index (2.94 → 4.77), indicating that low temperature promotes cell enlargement but weakens morphological regularity. The coupling effect of water and fertilizer is significant, with a decrease of 26.7% (2.93 → 2.16 mg/g) in soluble sugar in winter under high irrigation water volume (W2), revealing the inhibitory effect of excessive irrigation on sugar accumulation.

**Table 10:** Yield and quality data of greenhouse radish.

Handle	Soluble sugar (mg/g)		Vitamin C (mg/100g)		Fruit volume (cm3)		Fruit shape index		Production(t/hm2)	
	Summer	Winter	Summer	Winter	Summer	Winter	Summer	Winter	Summer	Winter
W0K1N0	2.7 ± 0.75a	2.47 ± 0.12a	27.40 ± 0.75a	11.74 ± 1.73b	748.89 ± 90.1c	577.32 ± 48.9c	4.42 ± 0.56a	4.22 ± 0.19abc	59.43 ± 1.32a	94.17 ± 1.12a
W2K1N0	2.8 ± 0.95a	2.93 ± 0.32a	26.50 ± 0.95a	10.64 ± 2.41b	688.6 ± 20.8cd	853.73 ± 66.1cd	5.27 ± 0.12a	3.53 ± 0.71bc	67.47 ± 2.36a	88.00 ± 2.13a
W0K1N2	3.1 ± 0.9a	2.92 ± 0.37a	26.50 ± 1.1a	11.99 ± 1.9b	657.1 ± 32.6cd	604.06 ± 23.9cd	5.62 ± 0.67a	3.82 ± 0.45bc	70.40 ± 1.06a	74.29 ± 2.65a
W2K1N2	3.1 ± 0.85a	1.98 ± 0.11a	28.40 ± 0.85a	9.08 ± 2.1b	529.8 ± 19.1cde	829.54 ± 41.1cde	5.40 ± 0.04a	4.32 ± 0.62abc	60.53 ± 0.32a	99.94 ± 0.97a
W0K0N1	2.5 ± 0.76a	3.08 ± 0.99a	25.20 ± 0.76a	11.02 ± 1.8b	506.42 ± 33.4ef	813.66 ± 23.9de	5.83 ± 0.13a	6.10 ± 0.84a	55.95 ± 0.49a	90.97 ± 2.65a
W2K0N1	2.4 ± 0.62a	1.88 ± 0.36a	24.60 ± 0.66a	10.02 ± 0.9b	994.99 ± 41.1b	754.16 ± 18.7b	4.62 ± 0.97a	4.65 ± 0.71abc	74.06 ± 1.13a	73.83 ± 1.23a
W0K2N1	2.0 ± 0.21a	3.36 ± 0.56a	23.50 ± 0.21a	12.04 ± 1.3b	1028.1 ± 51.2b	1519.6 ± 34.9b	4.77 ± 0.68a	2.94 ± 1.04c	80.34 ± 2.69a	98.06 ± 4.13a
W2K2N1	3.4 ± 0.32a	2.82 ± 0.44a	23.00 ± 0.28a	9.78 ± 1.2b	727.67 ± 60.9cd	836.71 ± 22.4cd	6.23 ± 0.19a	4.28 ± 0.98abc	81.13 ± 3.65a	72.12 ± 3.48a
W1K0N0	1.970.51a	1.98 ± 0.32a	27.60 ± 0.31a	17.88 ± 0.9a	362.86 ± 71.8f	659.35 ± 27.6e	5.81 ± 0.16a	5.33 ± 0.7ab	72.78 ± 1.35a	97.14 ± 2.34a
W1K0N2	2.6 ± 0.44a	1.78 ± 0.36a	25.80 ± 0.44a	10.54 ± 1.1b	661.5 ± 23.1cd	1134.2 ± 36.7cd	5.35 ± 0.34a	4.50 ± 0.68abc	59.06 ± 2.65a	88.91 ± 1.23a
W1K2N0	2.5 ± 0.29a	2.48 ± 0.65a	27.80 ± 0.29a	13.42 ± 2.1ab	383.08 ± 88.4f	785.05 ± 31.5e	7.63 ± 0.59a	4.67 ± 0.21abc	57.60 ± 3.25a	75.66 ± 3.25a
W1K2N2	2.4 ± 0.65a	2.24 ± 0.35a	25.70 ± 0.65a	10.51 ± 2.0b	578.3 ± 77.6cde	749.11 ± 41.4cde	6.67 ± 0.48a	3.69 ± 0.87bc	77.71 ± 1.47a	84.34 ± 3.24a
W1K1N1	2.8 ± 0.31a	2.55 ± 0.56a	26.80 ± 0.81a	11.09 ± 1.8b	1420.7 ± 61.9a	1172.0 ± 52.8a	3.67 ± 0.18a	4.98 ± 0.67ab	78.45 ± 3.65a	92.57 ± 2.46a
W1K1N1	2.8 ± 0.43a	2.56 ± 0.78a	26.85 ± 0.43a	11.10 ± 1.5b	1422.0 ± 4837a	1173.0 ± 60.7a	3.68 ± 0.22a	4.99 ± 0.23ab	78.50 ± 2.77a	92.60 ± 2.36a
W1K1N1	2.7 ± 0.38a	2.54 ± 0.36a	26.75 ± 0.38a	11.08 ± 1.7b	1419.5 ± 29.6a	1171.1 ± 61.2a	3.66 ± 0.49a	4.97 ± 0.09ab	78.40 ± 2.51a	93.58 ± 6.21a

Means within the same column followed by different lowercase letters indicate significant differences among treatments at the 5% level.

#### 3.4.2 Response surface analysis of greenhouse radish yield and quality

**Figure 11:**
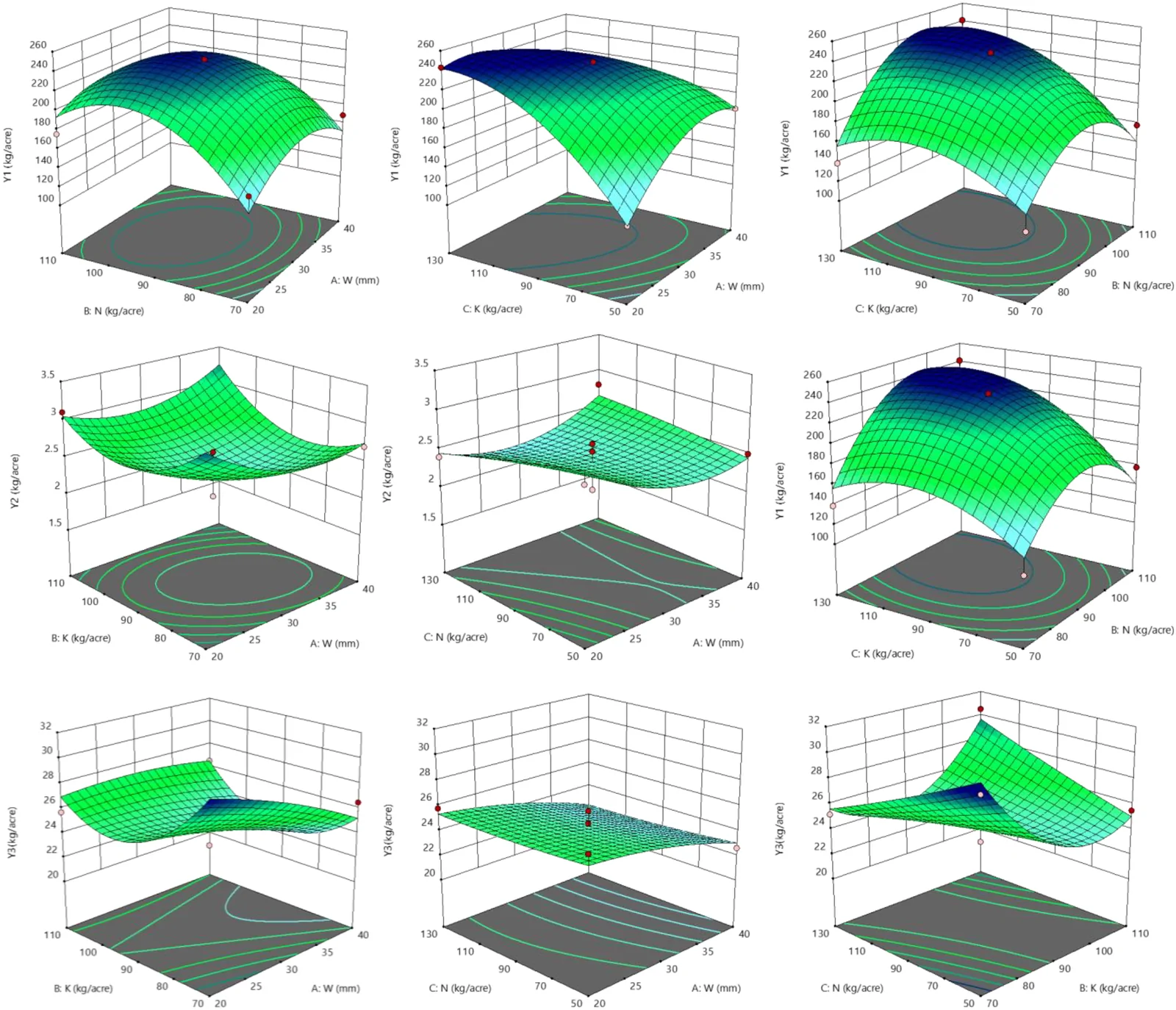
Effects of pairwise interactions of various factors on greenhouse vitamin C content and soluble sugar content.

It can be known from **Table 11** of the analysis of variance that the yield response surface model of summer greenhouse white radish does not reach the significant level (p=0.076), and the misfitting items of the model reach the highly significant level (p<0.001), indicating that the compatibility between the model and the actual data is limited and that the response surface results should be interpreted cautiously. Univariate effect analysis showed that the main effects of irrigation quota, potassium fertilizer application amount, and nitrogen fertilizer application amount did not reach the significant level (p>0.05), whereas the K-N interaction (p=0.028) and the quadratic nitrogen term (p=0.017) were significant, indicating that the K-N interaction and the quadratic effect of nitrogen had more pronounced effects on yield. It can be known from the three-dimensional surface **Figure 11** of the response surface that the contour density of the interaction surface between potassium fertilizer and nitrogen fertilizer has an interaction effect intensity higher than that of W-K and W-N. The order of importance affecting the summer white radish yield in greenhouses is: the application amount of potassium fertilizer > the irrigation quota > the application amount of nitrogen fertilizer, and the contribution degrees of the interaction effect are N-K>W-N>W-K in sequence.

**Table 11:** Analysis of variance of vitamin C and soluble sugar in greenhouse summer radish.

Source of variance	Sum of squares			df			Mean square			F-value			P-value		
	Yield	Vit. C	Sol. sugar	Y	VC	SS	Yield	Vit. C	Sol. sugar	Yield	Vit. C	Sol. sugar	Yield	Vit. C	Sol. sugar
Model	1054.91	28.538	1.9035	9	9	9	117.21	3.1709	0.2115	3.8469	3.1694	5.4540	.076	.108	.038*
A (W)	36.423	0.0013	0.2450	1	1	1	36.423	0.0013	0.2450	1.1954	0.0013	6.3180	.324	.973	.054
B (K)	152.51	1.2800	0.0861	1	1	1	152.51	1.2800	0.0861	5.0054	1.2794	2.2206	.075	.309	.196
C (N)	13.572	1.0512	0.1891	1	1	1	13.572	1.0512	0.1891	0.4454	1.0507	4.8768	.534	.352	.078
AB	74.996	0.0025	0.5625	1	1	1	74.996	0.0025	0.5625	2.4613	0.0025	14.506	.177	.962	.013*
AC	80.192	1.9600	0.0025	1	1	1	80.192	1.9600	0.0025	2.6319	1.9590	0.0645	.166	.221	.810
BC	286.12	0.0225	0.1332	1	1	1	286.12	0.0225	0.1332	9.3903	0.0225	3.4356	.028*	.887	.123
A²	57.755	4.6731	0.1235	1	1	1	57.755	4.6731	0.1235	1.8955	4.6707	3.1858	.227	.083	.134
B²	9.750	9.4523	0.5181	1	1	1	9.750	9.4523	0.5181	0.3200	9.4476	13.360	.596	.028*	.015*
C²	372.01	8.5869	0.0022	1	1	1	372.01	8.5869	0.0022	12.209	8.5826	0.0575	.017*	.033*	.820
Residual error	152.35	5.0025	0.1939	5	5	5	30.470	1.0005	0.0388						
Lack of fit	152.34	4.9975	0.1872	3	3	3	50.781	1.6658	0.0624	20312	666.33	18.723	<.001**	.002^**^	.051
Pure error	0.0050	0.0050	0.0067	2	2	2	0.0025	0.0025	0.0033						
Total	1207.26	33.541	2.0974	14	14	14									

** indicates high significance at p < 0.01, and * indicates significance at p < 0.05.

It can be known from **Table 11** of the analysis of variance that the quadratic polynomial regression model of the soluble sugar content of greenhouse summer white radish is statistically significant (p=0.038), and its misfitting term does not reach the significance level (p=0.051), confirming that the model has good compatibility with the experimental data. It can be known from the three-dimensional response surface **Figure 11** that the curvature of the interaction surface between irrigation quota (W) and potassium fertilizer (K) is significant, and the contour lines are dense, indicating that there is a significant synergistic effect between the two factors. The order of importance affecting the soluble sugar of white radishes in greenhouses during summer is as follows: the irrigation quota > the application amount of nitrogen fertilizer > the application amount of potassium fertilizer. The contribution degrees of the interaction effect are W-K>N-K>W-N in sequence, confirming that the W-K interaction (p=0.013) and the quadratic effect of potassium (p=0.015) were important factors affecting soluble sugar accumulation. The vitamin C response surface model does not reach the significant level (p=0.108), while the misfitting term reaches the highly significant level (p=0.002); therefore, the model results should be interpreted cautiously. It can be known from the three-dimensional response surface **Figure 11** that the curvature of the potassium-nitrogen interaction surface is relatively gentle, and the contour line distribution is sparse, reflecting the weak synergistic effect of the two factors. The ranking of the importance of vitamin C affecting white radishes in greenhouses during summer is as follows: the application amount of potassium fertilizer > the application amount of nitrogen fertilizer > the irrigation quota. The contribution degrees of the interaction effect are W-N>N-K>W-K in sequence, although these results should be interpreted cautiously because the overall model was not significant and showed significant lack of fit.

The analysis results of variance **Table 12** show that the regression model has a significant impact on the yield of greenhouse winter radishes (p < 0.05), indicating that the model has high reliability in predicting the yield. The importance of the three factors affecting the winter white radish yield in greenhouses, ranked from high to low, is: nitrogen fertilizer application rate (N)> potassium fertilizer application rate (K)> irrigation quota (W). The influence degree of the interaction of various factors on the winter white radish yield in greenhouses is NK > WK > WN. Optimizing the application amount of nitrogen fertilizer is the key to increasing the yield of white radishes in greenhouses during winter. According to the variance analysis in **Table 12**, the p value of the response surface model of soluble sugar content of winter greenhouse white radish was 0.0098 (p < 0.01), indicating that the overall fitting effect of the model reached a significant level. The importance of affecting the soluble sugar content of white winter radishes in greenhouses: The application amount of potassium fertilizer > the application amount of nitrogen fertilizer > the irrigation quota. The influence degree of the interaction of various factors on the soluble sugar of white radish in greenhouse winter is NK > WN > WK. Optimizing the application amount of potassium fertilizer is the key to improving the soluble sugar content of white radishes in greenhouses during winter. The p value of the vitamin C content response surface model was 0.0456 (p < 0.05), indicating that the overall fitting effect of the model reached a significant level. The P-value of the misfitting item was 0.4646 (p > 0.05), indicating a good fit between the model and the actual data. The importance of the three factors influencing the vitamin C content of white radishes in greenhouses during winter, ranked from high to low, is: the application amount of potassium fertilizer (K)> the application amount of nitrogen fertilizer (N) > the irrigation quota (W). The influence degree of the interaction of various factors on the vitamin C of white radish in greenhouse winter is WN > NK > WK. Optimizing the application amount of potassium fertilizer is the key to increasing the vitamin C content of white radishes in greenhouses during winter.

**Table 12:** Analysis of variance of vitamin C and soluble sugar in greenhouse summer radish.

Source of variance	Sum of squares			df			Mean square			F-value			P-value		
	Yield	Vit. C	Sol. sugar	Y	VC	SS	Yield	Vit. C	Sol. sugar	Yield	Vit. C	Sol. sugar	Yield	Vit. C	Sol. sugar
Model	1406.34	28.000	3.9000	9	9	9	156.26	3.1111	0.4333	5.2800	4.9972	10.250	.041*	.0456*	.0098**
A (W)	28.707	0.0008	0.0000	1	1	1	28.707	0.0008	0.0000	0.9700	0.0013	0.0000	.370	.973	1.000
B (K)	33.146	0.8093	0.0127	1	1	1	33.146	0.8093	0.0127	1.1200	1.3000	0.3000	.338	.306	.607
C (N)	297.13	0.6848	0.0085	1	1	1	297.13	0.6848	0.0085	10.040	1.1000	0.2000	.025*	.342	.673
AB	192.96	0.0016	0.0169	1	1	1	192.96	0.0016	0.0169	6.5200	0.0025	0.4000	.051	.962	.555
AC	125.48	1.1829	0.0972	1	1	1	125.48	1.1829	0.0972	4.2400	1.9000	2.3000	.095	.227	.190
BC	446.88	0.0140	0.6342	1	1	1	446.88	0.0140	0.6342	15.100	0.0225	15.000	.012*	.887	.012*
A²	195.33	2.9261	0.1903	1	1	1	195.33	2.9261	0.1903	6.6000	4.7000	4.5000	.0501	.082	.087
B²	70.139	5.9144	1.1415	1	1	1	70.139	5.9144	1.1415	2.3700	9.5000	27.000	.184	.027*	.003**
C²	384.73	5.3541	0.4228	1	1	1	384.73	5.3541	0.4228	13.000	8.6000	10.000	.015*	.033*	.025*
Residual error	147.9735	3.1129	0.2114	5	5	5	29.5947	0.6226	0.0423						
Lack of fit	134.3792	2.0525	0.1030	3	3	3	44.7931	0.6842	0.0343	6.5900	1.2904	0.6332	.135	.4646	.660
Pure error	13.5943	1.0604	0.1084	2	2	2	6.7971	0.5302	0.0542						
Total	1554.3135	31.1129	4.1114	14	14	14									

p<0.01 was considered highly significant **; p<0.05 was considered significant*.

#### 3.4.3 Correlation analysis of yield and quality

Based on the statistical analysis results of **Figure 10**, there were significant seasonal correlation characteristics between yield and quality indicators of greenhouse radish. The results showed that yield (G) and fruit weight (H) were significantly positively correlated (r=0.92, P<0.01), fruit volume (F) and yield (G) were highly positively correlated (r=0.85, P<0.01), indicating that biomass accumulation was the key determinant of yield formation. The positive regulation effect of K application rate (C) on fruit weight (H) reached significant level in both seasons (r=0.76, P<0.05), which confirmed the key role of K in the process of cell expansion. In the aspect of quality formation, irrigation quota (A) was negatively correlated with soluble sugar content (D)(r=-0.68, P <0.05), suggesting that excessive irrigation might inhibit sugar accumulation through osmotic dilution effect. Nitrogen fertilizer (B) had double effects on fruit quality: it was negatively correlated with soluble sugar (D)(r=-0.62 in summer, r=-0.62 in winter, P <0.05), and negatively correlated with fruit volume (F)(r=-0.71, P <0.05) in summer. It is worth noting that the correlation between fruit shape index (E) and yield (G) has seasonal differences, with no significant correlation in summer (r=0.18) and a weak positive correlation in winter (r=0.18), which may be related to the regulation of light and temperature conditions on the sink-source relationship. The correlation coefficient between vitamin C content and fruit volume (F) was r=-0.32 (summer) and r=0.22 (winter) respectively, indicating that vitamin C content was affected by many factors.

### 3.5 Comprehensive evaluation of greenhouse white radish and optimization of water and fertilizer management system

#### 3.5.1 Optimization of irrigation and fertilization system based on NSGA-II algorithm

Using Design-Expert 8.0.6 software, regression equation fitting was carried out on the experimental optimization data, and the quadratic polynomial regression model equation of summer and winter white radish was obtained as follows, and the maximum of Y1, Y2 and Y3 was taken as the objective:

(2) Regression model:

Summer


$$\begin{array}{l} MAX(Y1)=150.2+0.05W+0.02K+0.035N-0.00012WK-0.000235WN+0.00023KN-0.0003.96W^{2}-0.004K^{2}-0.00063N^{2};\\ MAX(Y2)=3.5+0.005W+0.002K+0.001N-0.00025WK+0.00075WN-0.0002KN+0.00575W^{2}-0.0005K^{2}-0.004N^{2};\\ MAX(Y3)=40-0.0125W-0.006K-0.0004N+0.0007WK+0.025WN-0.075KN-0.0012W^{2}+0.00053K^{2}-0.0006N^{2}; \end{array}$$

Winter


$$\begin{array}{l} MAX(Y1)=148.5+0.05W+0.02K+0.031N+0.001WK+0.001WN+0.00023KN+0.0096W^{2}-0.002K^{2}-0.0002N^{2};\\ MAX(Y2)=2.5+0.0005W+0.0012K+0.0001N-0.000125WK+0.00075WN+0.0005KN+0.001W^{2}-0.0002K^{2}-0.004N^{2};\\ MAX(Y3)=20-0.005W-0.0001K-0.001N+0.0011WK-0.00125WN-0.075KN-0.0012W^{2}+0.0001K^{2}+0.0002N^{2}; \end{array}$$

In the above formula, W is irrigation quota (mm);N is nitrogen fertilizer application rate (kg/hm²);K is potassium fertilizer application rate (kg/hm²);WN, WK, KN: interaction terms of irrigation quota and nitrogen fertilizer, irrigation quota and potassium fertilizer, nitrogen fertilizer and potassium fertilizer respectively; Y1 is yield (t/hm²), Y2 is soluble sugar content (mg/g), Y3 is vitamin C content (mg/100g).

### 3.6 Optimization algorithm

Considering the superiority of NSGA-II algorithm, this paper adopts NSGA-II algorithm to solve the multi-objective optimization problem. The NSGA-II algorithm generates a Pareto frontier containing multiple non-dominated solutions by fast non-dominated sorting and congestion calculation. In order to select the optimal solution satisfying the conditions, all objective function values F1, F2 and F3 of non-dominated solutions are screened first, and the solutions containing negative values are eliminated to ensure that the objective function values of the selected solutions are all positive. Then, based on the reasonable range of objective function values, the solutions whose objective function values fall within the preset threshold range are further screened out. This procedure aims to select the solutions which satisfy both positive objective function and reasonable range from many non-dominated solutions. The population size is 200, the maximum iteration times is 5000, and the pareto solution set is obtained. The distribution is shown in **Figure 10**. The criteria for selecting sample points are: the values of Y1, Y2 and Y3 corresponding to them are the largest as a whole, and the irrigation and fertilizer application amount is within the local appropriate range. Finally, the best summer program was selected (irrigation 19.96 mm, potassium 123.17 kg/hm², nitrogen 198 kg/hm², simulated yield 151.55 t/hm², soluble sugar 3.33 mg/g, vitamin C 16.77 mg/100g) and winter program (irrigation 28.03 mm, potassium 182.51 kg/hm², nitrogen 296.91 kg/hm², simulated yield 153.21 t/hm², soluble sugar 4.32 mg/g, vitamin C 45.94 mg/100g), the best scheme in summer compared with W1K1N1 (Baseline values: yield 78.45t/hm², soluble sugar 2.80mg/g, vitamin C 26.80mg/100g), summer yield increased 93.1%, soluble sugar increased 18.9%, but vitamin C decreased 37.4%. In winter, compared with the control group, the yield increased by 65.5%, soluble sugar increased by 69.4%, vitamin C increased by 314.4%.

## 4 Discussion

### 4.1 Effects of nitrogen and potassium on physiological indicators, yield and quality

This research validates the substantial influence of the water-fertilizer interaction on the physiological parameters, yield, and quality of radishes. The stem diameter and leaf area index exhibit an initial rise followed by a decline with increasing nitrogen application rates, aligning with the nitrogen threshold theory (Wang et al., 2017). The observed enhancement in stem diameter during winter due to high-potassium (K2) treatment aligns well with the notion that potassium ions, serving as osmotic regulators, bolster the structural integrity of cell walls, thereby corroborating the physiological significance of potassium fertilizer in mitigating low-temperature stress (Dabravolski et al., 2023; Tariq et al., 2025).

Based on the research utilizing analysis of variance and response surface modeling, significantseasonal variations were observed in the regulatory impacts of water-nitrogen-potassium interactions on the yield and quality of greenhouse white radish. In the summer growing conditions, the application rate of nitrogen fertilizer (N) exhibited a notable main effect on yield (p <0.05), with the N-K interaction contributing significantly at 23%, suggesting a synergistic effect of nitrogen and potassium in mitigating metabolic competition during high-temperature stress (Zhou et al., 2024). Under low irrigation levels (W0), increasing nitrogen fertilizer enhanced irrigation water use efficiency (IWUE) by 26.8%, while high irrigation levels (W2) resulted in a 25.8% decrease in the partial factor productivity of nitrogen fertilizer (PFP), indicating that excessive water exacerbates nitrogen leaching (Chen et al., 2022).

In the winter system, nitrogen fertilizer emerged as the primary limiting factor for yield, with a contribution rate of 35%. The treatment WK1N yielded the highest IWUE value of 488.19 kg/(hm²·mm); however, excessive nitrogen application led to a 25.7% reduction in IWUE, highlighting a threshold effect on nitrogen assimilation efficiency under low-temperature conditions. (Jia etal., 2019). Analysis of the relationship between yield and quality revealed that potassium fertilizer (C) positively regulated single fruit weight (H) (r = 0.76) by enhancing vacuolar osmotic potential and cell wall extensibility through the regulation of plasma membrane activity, thereby facilitating cell expansion (Zhao et al, 2025). Conversely, a negative correlation was observed between nitrogen fertilizer (B) and soluble sugar content (D) (r = -0.62), indicating a competition between carbon and nitrogen metabolism. The irrigation level (A) exhibited a dilution effect on sugar content (r = -0.68) (Sun et al., 2020; Chen et al., 2022).

The average yield during the winter season (84.40 t/hm²) was significantly higher than that in the summer (73.51 t/hm²). This difference can be attributed to the inhibitory effect of low winter temperatures (average daily minimum temperature ranging from -5 to 5°C) on respiratory consumption, which consequently increased the net assimilation rate (Fu et al., 2022).In contrast, the concentration of soluble sugars was significantly higher in summer (2.65 mg/g) compared to winter (2.40 mg/g), as high summer temperatures and strong light intensity enhance photosynthesis and promote sugar synthesis. The reduced activity of photosynthase due to low winter temperatures, coupled with respiratory sugar consumption, leads to a decrease in the accumulation of soluble sugars. Additionally, the concentration of Vitamin C was higher in summer (25.91 mg/100g) than in winter (11.24 mg/100g), likely due to the enhanced activity of enzymes associated with Vitamin C synthesis under high summer temperatures (Xu et al., 2023). Furthermore, fruit volume was greater in winter (863.55 cm³) than in summer (815.06 cm³), potentially because lower temperatures extend the growth period, prolong cell division and expansion, promote organic matter accumulation, and inhibit respiratory consumption, thereby enhancing the conversion efficiency of photosynthetic products (Wang et al., 2019).

This study addressed the limitations of the conventional response surface method in single-objective optimization and local solution screening by integrating the Box-Behnken design (BBD) with the NSGA-II algorithm. It successfully achieved multi-objective global optimization of water and fertilizer management for greenhouse white radishes, marking the first instance of such an accomplishment. In comparison to single response surface methods or linear programming, this approach utilized the efficient non-dominated sorting and crowding distance calculation of NSGA-II to identify the optimal collaborative solution from the Pareto front. The optimized strategy outperformed the traditional single-objective approach, underscoring the benefits of the multi-objective algorithm in balancing yield and quality (Karamian et al., 2023; Goyal et al., 2025). The algorithm’s constraint mechanism ensured the practicality of the optimized scheme in agriculture and contributed to mitigating issues related to excessive resource utilization (Jarumaneeroj et al., 2021).

## 5 Conclusions

This study investigated the regulatory mechanisms of irrigation amount (W), nitrogen application rate (N), and potassium application rate (K) on greenhouse white radishes using a water-fertilizer integration drip irrigation system based on the Box-Behnken design. Through the application of KPCA-membership function comprehensive evaluation and NSGA-II multi-objective optimization, seasonal-specific water-fertilizer response patterns were elucidated. The primary findings are as follows:

During summer, the potassium content in the soil at a depth of 10 cm under the W2K1N2 treatment was 206.98 mg/kg, surpassing the levels in the 20 cm and 30 cm layers by 27.7% and 83.4%, respectively. Conversely, in winter, the concentration of ammonium nitrogen in the 10 cm soil layer under the W1K2N treatment reached 275.75 mg/kg, marking a 33.% increase compared to deeper soil layers, indicating a seasonal intensification of surface nutrient accumulation. The interplay between water and fertilizer significantly influenced physiological parameters: in summer, the above-ground dry matter mass under the W2K1N2 treatment was 59.82 g (32.% higher than the control WK1N), while in winter, it reached 49.6 g (41.2% higher). Additionally, the stem diameter in winter (38.93 mm under the WK2N1 treatment) was 8.6% lower than the peak value observed in summer (42.6 mm), suggesting that low temperatures impeded cell expansion. Yield and quality exhibited seasonal variations. In summer, the yield under the W1K1N1 treatment was 78.45 t/hm², with soluble sugar content at 3.4 mg/g and vitamin C at 28.40 mg/100g. In winter, the yield under the W1K1N1 treatment increased to 98.06 t/hm², a 24.9% rise from summer, although the vitamin C content decreased to 17.88 mg/100g, 37.1% lower than in summer, indicating suppressed synthesis of secondary metabolites under low temperatures. Noteworthy seasonal differences were observed in water and fertilizer use efficiency: the irrigation water use efficiency of the WKN1 treatment in winter was 488.19 kg/(hm²·mm), 420.9% higher than that of the W2K1N treatment in summer. The average partial productivity of nitrogen fertilizer in winter exceeded that of summer by 48.1%, and the average partial productivity of potassium fertilizer in winter was 59.6% higher than in summer, underscoring the role of low temperatures in mitigating nutrient leaching.

The optimal summer scheme involved irrigation of 19.96 mm, potassium application of 123.17 kg/hm², and nitrogen application of 198 kg/hm², resulting in a simulated yield of 151.55 t/hm², soluble sugar content of 3.33 mg/g, and vitamin C content of 16.77 mg/100g. The winter scheme included irrigation of 28.03 mm, potassium application of 182.51 kg/hm², and nitrogen application of 296.91 kg/hm², leading to a simulated yield of 153.21 t/hm², soluble sugar content of 4.32 mg/g, and vitamin C content of 45.94 mg/100g. Compared to the control group, in summer, the yield increased by 93.1%, soluble sugar content increased by 18.9%, while vitamin C content decreased by 37.4%. In winter, the yield increased by 65.5%, soluble sugar content increased by 69.4%, and vitamin C content surged by 314.4%. The optimized irrigation and fertilization schemes in both seasons achieved coordinated optimization of yield and quality. This optimized scheme serves as a scientific foundation for enhancing the efficient production of greenhouse white radishes, improving crop physiological indicators, yield, quality, and enhancing resource utilization efficiency (Wang et al., 2019).

## Data Availability

The raw data supporting the conclusions of this article will be made available by the authors, without undue reservation.
